# A Luminescent Pt-NCN Complex Anchored onto a Polyethylene Film for Sequential Detection of Iodide and Mercury

**DOI:** 10.1021/acsomega.6c08055

**Published:** 2026-09-28

**Authors:** Josue Valdes-García, Mireille Vonlanthen, Fabián Cuétara-Guadarrama, Ernesto Rivera, Roxana Marisol Calderón-Olvera, Juan Carlos Alonso-Huitrón, David Garcia-Bassoco, Hector Luis Valdés-Negrín, Guillermina Burillo, Alejandro Dorazco-González

**Affiliations:** † 7180 Instituto de Investigaciones en Materiales, Universidad Nacional Autónoma de México, Circuito Exterior, Ciudad Universitaria, Ciudad de Mexico C.P. 04510, México; ‡ Instituto de Ciencias Nucleares, Universidad Nacional Autónoma de México, Circuito Exterior, Ciudad Universitaria, Ciudad de Mexico C.P. 04510, México; § Instituto de Química, Universidad Nacional Autónoma de México, Circuito Exterior, Ciudad Universitaria, Ciudad de Mexico C.P. 04510, México

## Abstract

Luminescent polymers have attracted attention as solid-state sensing platforms for selective analyte recognition; however, the use of [Pt­(NCN)­Cl]-based polymers for chemosensing remains largely unexplored. Herein, luminescent platforms (**PE-** ***g*** **-MC-1-X**) based on a [Pt­(NCN)­Cl] complex (NCN = 1,3-bis­(benzimidazol-2-yl)­benzene derivative bearing two tetraethylene glycol (TEG) chains) (**1**) anchored onto polyethylene grafted with methacryloyl chloride (**PE-** ***g*** **-MC**) by gamma irradiation at different doses (**X** = 2, 4, 6, and 8 kGy) were synthesized and characterized. Among luminescent platforms, **PE-** ***g*** **-MC-1-8kGy** film was studied as a chemosensor for anions, nucleotides, and oxyanions in ethanol/water (1:1, v/v). The luminescent polymer exhibited a selective quenching response toward iodide (I^–^), with a quenching constant (*K_q_*) of 4.67(±0.49) × 10^3^ M^–1^ and a limit of detection of 6.55(±0.26) ppm. Based on analyses by attenuated total reflectance Fourier-transform infrared (ATR-FTIR) spectroscopy, scanning electron microscopy coupled with energy-dispersive X-ray spectroscopy (SEM-EDS), and X-ray photoelectron spectroscopy (XPS), the binding mode of iodide was attributed to its accumulation within hydrophobic domains of TEG chains through an ion-specific effect, perturbing the electronic environment of the [Pt­(NCN)­Cl] unit. Sequential addition of mercury (Hg^2+^) in the presence of hydrochloric acid (HCl) restored the emission intensity, with a limit of detection of 2.40(±0.11) ppm. The luminescent material exhibited reversibility and reusability over multiple I^–^/Hg^2+^ sensing cycles. These results confirm that immobilization of the Pt-NCN complex onto a polymer matrix functionalized by gamma irradiation provides a solid-state sensing platform for the sequential detection of I^–^ and Hg^2+^.

## Graphical abstract

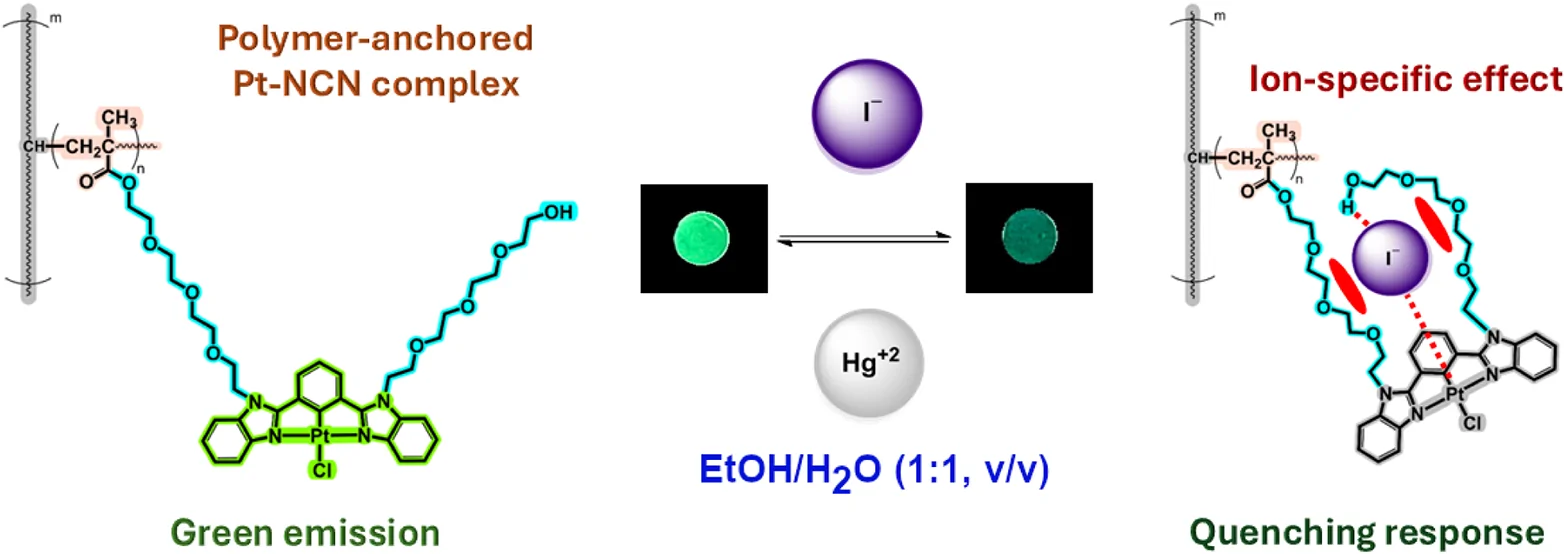

## 1 Introduction

Selective recognition of iodide (I^–^) and mercury (Hg^2+^) remains an active area of research owing to their industrial, environmental and biological relevance.^1–4^ Iodide plays a crucial role in biological processes, including thyroid function, neurological development, and cell growth,^5–8^ while an imbalance in iodide levels is associated with disorders such as hyperthyroidism, hypothyroidism, thyroiditis, and goiter.^6,9–12^ In industry, I^–^ is used in dietary supplements, X-ray contrast agents,^13–15^ antiseptics,^16,17^ and organic synthesis.^18,19^ However, its widespread use can lead to its release into the environment as industrial waste, raising environmental concerns.^14,15^ Among hazardous environmental contaminants, mercury is considered one of the most dangerous heavy metal pollutants due to its high toxicity, persistence and bioaccumulation.^20,21^ Hg^2+^ toxicity arises from its strong affinity for sulfur–containing biomolecules, disrupting essential biological processes and leading to severe neurological and renal damage.^22–29^ Therefore, the development of selective and sensitive chemosensors for iodide and mercury detection is highly desirable.

Luminescent chemosensors are particularly attractive owing to their high sensitivity, low cost, real–time visual detection, and ease of operation.^30–32^ In these systems, I^–^ detection has been achieved through hydrogen bonding,^33–35^ halogen bonding,^36,37^ electrostatic interactions,^38,39^ and coordination to metal centers.^40,41^ Typically, metal–based chemosensors operate through sequential sensing, where mercury,^42–44^ silver^45,46^ or copper^43,47,48^ are first recognized and subsequently displaced by iodide. However, platinum–based systems remain scarcely explored, despite platinum’s affinity for I^–^ predicted by the hard–soft–acid–base (HSAB) principle.^49,50^ In this context, Masih and coworkers reported a platinum porphyrin complex that exhibited a turn–off response toward I^–^.^51^ The quenching response was attributed to an axial electron–transfer process involving I^–^ and the Pt center in the excited state. However, its selectivity was limited because bromide induced a quenching response comparable to that of iodide. On the other hand, Pt-NCN complexes possess tunable photophysical properties arising from modifications of the ligand structure and the Pt coordination environment.^52–54^ These compounds generally display structured emission bands at 500–650 nm, attributed to mixed intraligand (^3^IL) and metal–to–ligand charge–transfer (^3^MLCT) excited states.^55–57^ Although cationic Pt-NCN complexes have mainly shown selectivity toward Cl^–^ through the cooperative hydrogen bonding and coordination at the Pt center,^58,59^ Pt–I interactions have also been demonstrated in Pt-NCN crystals.^57–60^ Moreover, computational studies have revealed axial coordination of I_2_ to these complexes,^61,62^ highlighting the potential of Pt-NCN complexes for selective I^–^ detection.

Beyond molecular chemosensors, polymeric sensing platforms have also attracted attention for anion detection, as their surface properties contribute to selective recognition through ion–specific effects.^63,64^ Chaotropic ions, such as iodide, preferentially accumulate at hydrophobic polymer interfaces, where disruption of the bulk water hydrogen–bonding network is energetically more favorable than the simultaneous hydration of both the chaotropic ions and the polymer surface.^65–67^ In this context, selective adsorption of chaotropic anions has been reported for several methyl–rich polymers, including poly­(propylene oxide),^63^ poly­(*N*-isopropylacrylamide),^63^ poly­(methyl methacrylate),^68^ and polydimethylsiloxane.^68^ This behavior has been attributed to enhanced London dispersion forces between large anions and electron–deficient methyl groups.^68^ Similarly, poly­(ethylene oxide) oligomers tend to accumulate chaotropic anions within the center of the macromolecule chain.^69^

In this regard, poly­(3-alkoxy-4-methylthiophene) exhibited a bathochromic–shift in absorption upon I^–^ binding, attributed to electrostatic interactions and the affinity of I^–^ for the polymer’s apolar domains.^70^ On the other hand, a thiazole–containing conjugated polymer exhibited a turn–off response and color change from colorless to yellow upon addition of I^–^, attributed to interaction with the thiazole–containing polymer chains.^71^ The original optical properties were restored after subsequent addition of Hg^2+^, enabling sequential I^–^/Hg^2+^ sensing. However, the luminescent polymer exhibits blue emission, whose optical response can be affected by light scattering and background fluorescence. Considering these findings, the incorporation of a Pt-NCN complex into a polymeric platform could overcome these drawbacks by combining the properties of both components. Pt-NCN complexes have been immobilized on solid supports, including silica,^72,73^ polyglycerol,^74,75^ and nanostructured architectures,^76,77^ for applications in catalysis. Nevertheless, their incorporation into polymeric matrices for luminescent chemosensing remains largely unexplored.

One strategy to immobilize chemosensors onto polymer surfaces involves their covalent attachment to functionalized polymers. Surface functionalization by gamma irradiation enables the grafting of reactive monomers, such as acryloyl chloride or methacrylic acid, through radical pathways.^78–83^ Acyl chloride moieties allow covalent anchoring of metal complexes via linker or spacer molecules.^84^ Linker molecules such as tetraethylene glycol,^78–81^ 1,8-diaminododecane,^85^ and cysteamine,^86^ can also modulate hydration capacity,^87^ analyte diffusion,^88^ steric environment,^89^ and local polarity,^70^ thereby influencing sensing performance.

Herein, we report a series of luminescent polymer platforms based on a Pt-NCN complex (**1**) anchored onto a functionalized polyethylene irradiated at different doses (**PE-g** **-MC-1-X**, **X** = 2, 4, 6, or 8 kGy). The synthesis, characterization and optical properties of luminescent polymers are reported. Among them, **PE-g** **-MC-1-8kGy** was used as a chemosensor for the selective detection of I^–^ and Hg^2+^. Fluorescence recognition studies revealed that **PE-g** **-MC-1-8kGy** detects I^–^ via an emission quenching response, whereas the subsequent addition of Hg^2+^ restores the emission intensity. The apparent quenching constant (*K_q_*) and the accessible fraction of receptor **1** (*f_a_*) on the polymer surface were determined. In addition, **PE-g** **-MC-1-8kGy** was applied to the quantification of I^–^ in a real dietary supplement sample. The limits of detection (LOD) for I^–^ and Hg^2+^ were calculated. The interaction of I^–^ within the luminescent polymer was supported by attenuated total reflectance Fourier–transform infrared (ATR-FTIR) spectroscopy, scanning electron microscopy coupled with energy–dispersive X-ray spectroscopy (SEM-EDS), X-ray photoelectron spectroscopy (XPS), and Stern–Volmer quenching studies. Finally, the reversibility and reusability of the material were demonstrated over multiple sensing cycles.

## 2 Experimental Section

### 2.1 General Conditions

A commercial polyethylene (**PE**) film (PEMEX, México) with a density of 0.926 g/mL, a crystallinity of 62%, and a thickness of 0.07 mm was used in this work. The film was cut into 1 × 5 cm pieces, washed with methanol (MeOH) overnight, and dried under vacuum until constant weight. Methacryloyl chloride (MC), purchased from Sigma–Aldrich, was distilled before use. The distillation was carried out with safety precautions due to its toxic and lachrymatory nature. Anhydrous toluene, dichloromethane (DCM), MeOH and triethylamine (Et_3_N) were used as received from Sigma–Aldrich. Nuclear magnetic resonance (^1^H and ^13^C NMR) spectra of the characterization were recorded on a Bruker Advance DPX 300 spectrometer, operating at 300 and 75.5 MHz for ^1^H and ^13^C, respectively. Electrospray ionization mass spectrometry (ESI-MS) spectrum in positive–ion mode was recorded on an Agilent Tech 6530BA Q-TOF LC/MS spectrometer. ATR-FTIR spectra were recorded on a Nicolet iS50 FTIR spectrometer. Elemental analysis (EA) was performed using a Thermo Scientific Flash 2000 instrument at 950 °C. Thermogravimetric analysis (TGA) was recorded on a TGA Q500 Instrument from 50 to 700 °C at a heating rate of 10 °C min^–1^. Differential scanning calorimetry (DSC) measurements were performed using a TA Instrument Q2000 differential scanning calorimeter from 20 to 200 °C at a heating rate of 10 °C min^–1^. Atomic force microscopy (AFM) measurements were performed using a JEOL JSPM-4210 atomic force microscope. SEM-EDS images were acquired using a JEOL 7600F Scanning Electron Microscope. XPS measurements were performed using a Physical Electronics PHI 5000 Versa-Probe II scanning XPS microprobe system under ultrahigh vacuum conditions. The chemical composition and oxidation states of the elements present in the samples were investigated by XPS. The XPS spectra were analyzed using MultiPak and SDP v4.1 software. UV-vis spectra were recorded as thin plastic films on a Shimadzu U-260 spectrophotometer. Fluorescence spectra were recorded on a Fluorolog 3 spectrofluorometer (Horiba) equipped with a xenon lamp as the light source.

### 2.2 Synthesis of Pt-NCN Complex, **1**

Compound **1** was synthesized following the procedure previously reported by our research group.^90^ Spectroscopy data were consistent with those previously reported (Figures S1–S4). Yield 78% (57.2 mg). ^**1**^ **H NMR** (300 MHz, 298 K, CDCl_3_, δ): 8.83–8.80 (m, 2H), 7.38 (dd, *J* = 7.7, 3.8 Hz, 2H), 7.28 (m, overlapped with residual CHCl_3_, 2H), 7.20 (m, 4H), 7.03 (t, *J* = 7.7 Hz, 1H), 4.64 (t, *J* = 5.5 Hz, 4H), 3.85 (t, *J* = 5.4 Hz, 4H), 3.61 (m, 4H), 3.49–3.28 (m, 22H). ^**13**^ **C NMR** (75.5 MHz, 298 K, CDCl_3_, δ): 161.5, 161.0, 141.2, 134.4, 131.6, 124.5, 124.3, 123.1, 122.6, 119.1, 109.8, 72.5, 71.1, 70.6, 70.5, 70.2, 69.4, 61.6, 45.0. **ESI­(+)-MS** (m/z): calculated for C_38_H_49_N_5_O_8_Pt [**1** – Cl^–^ + MeCN]^+^, 898.91 m/z; found 898.30 m/z. **ATR-FTIR** (cm^–1^): ν = 3391.40 (w), 2863.77 (m), 1441.38 (m), 1096.75 (s), 745.84 (s), 721.74 (s), 437.67 (w). Elemental analysis calculated for C_36_H_45_ClN_4_O_8_Pt (%): C, 48.46; H, 5.08; Cl, 3.97; N, 6.28; O, 14.34; Pt, 21.86. Found: C, 47.83; H, 4.90; N, 5.77.

### 2.3 Grafting **MC** onto **PE** Films (**PE-g-MC**)

**PE** films were placed individually in Pyrex ampules containing 7 mL of a dry MC/toluene solution (50% v/v). The ampules were degassed under vacuum using freeze–pump–thaw cycles. Afterward, the ampules were sealed and irradiated with a ^60^Co γ–source (Gammabeam 651 PT, Nordion International Inc.) at a dose rate of 10.32 kGy h^–1^, yielding two **PE-g-MC** films irradiated at doses of 2, 4, 6, and 8 kGy. After irradiation, the **PE-g-MC** films were promptly and carefully removed from the ampules and placed in dry DCM in closed vials, where they were stirred overnight. After stirring, the **PE-g-MC** films were used immediately for the anchoring of complex **1** and for the esterification with MeOH, as described below.

### 2.4 Anchoring of Complex **1** onto **PE-g-MC** (**PE-g-MC-1**)

One **PE-g-MC** film from each irradiation dose was immersed in a solution of **1** (3 mM) in dry DCM (7 mL) containing Et_3_N (1%) and stirred overnight. Subsequently, the resulting films (**PE-g** **-MC-1**) were washed overnight with dry MeOH to convert the remaining chloride into the methyl ester. Finally, the **PE-g** **-MC-1** films were dried under vacuum until constant weight. The remaining **PE-g-MC** films were immersed in dry MeOH containing Et_3_N (1%) and stirred overnight to complete esterification. The resulting films (**PE-g** **-MC-MeO**) were washed with MeOH to remove Et_3_N and dried under vacuum until constant weight. Equation 1 was used to estimate the weight–based grafting yield:

1 $$Grafting\;\left(\%\right)=100\left(W-W_{0}\right)/W_{0}$$

where *W* is the weight of **PE-g** **-MC-MeO** film and *W*_0_ corresponds to the weight of **PE** film before grafting. The percentage in weight of the incorporated compound **1** was determined using the weights of the **PE-g** **-MC-1** and **PE-g** **-MC-MeO** films at the same irradiation dose, as shown in eq 2:

2 $${Anchoring}_{complex\;1}\left(\%\right)=100\left(W_{1}-W_{MeOH}\right)/W_{0}$$

where *W*_***1***_ and *W*_*MeOH*_ are the weights of the **PE-g** **-MC-1** and **PE-g** **-MC-MeO** films, respectively, and *W*_0_ is the weight of the **PE** film before grafting. The **PE-g-MC** films were not used as references due to their reactivity to ambient humidity. For that reason, **PE-g** **-MC-MeO** films were used, as they are more stable and exhibit weights comparable to those of the chloride–containing films.

### 2.5 Analysis of a KI Dietary Supplement

The applicability of the proposed luminescent sensing platform was further evaluated through the analysis of a commercially available KI dietary supplement. One tablet was ground in a mortar to obtain a fine powder. Afterward, 7.4 mg of powder was dispersed into 10 mL of Milli–Q water. The resulting solution was sonicated for 10 min, filtered and diluted to a final volume of 15 mL in a volumetric flask. An aliquot of 5 mL of that solution was mixed with 5 mL of EtOH containing a circular **PE-g** **-MC-1-8kGy** sample and stirred for 1 h. Emission spectra were recorded before and after exposure.

### 2.6 Reusability of the **PE-g-MC-1-8kGy** Sensing Platform

The reusability of the proposed sensing platform was evaluated through successive iodide sensing and mercury–induced emission recovery cycles. Three circular **PE-g** **-MC-1-8kGy** samples were used for the experiments. Each sample was immersed in a solution of NaI (45 ppm) in EtOH/H_2_O (1:1, v/v) for 1 h. Afterward, the circular sample was blotted on absorbent paper, and its emission spectrum was recorded. Subsequently, it was immersed in a solution of HgCl_2_ (23 ppm) containing one drop of HCl (37 wt %) in EtOH/H_2_O (1:1, v/v) for 1 h. The circular sample was then removed from the solution, washed with distilled water, blotted on absorbent paper and its emission spectrum was recorded again.

## 3 Results and Discussion

### 3.1 Synthesis of **PE-g-MC-1** Films

The **PE-g** **-MC-1** films were synthesized by the anchoring method, as depicted in Scheme 1. **PE** films immersed in a dry MC/toluene solution (50% v/v) were irradiated with a ^60^Co γ–source for different irradiation times to obtain **PE-g-MC** films at doses of 2, 4, 6, and 8 kGy. Finally, the anchoring of compound **1** onto the **PE-g-MC** films was performed by immersion of each **PE-g-MC** film in dry DCM containing compound **1** (3 mM) and Et_3_N (1%), yielding the corresponding **PE-g** **-MC-1** film. The grafting and compound **1** anchoring percentages (% weight) of each film are listed in Table 1. Among the obtained **PE-g** **-MC-1** films, **PE-g** **-MC-1-8kGy** film exhibited the highest grafting (28.22%) and anchoring (2.65%) percentages. However, these values are lower than those reported for **PE** films grafted with acryloyl chloride.^78–81^ This difference can be attributed to the methacryloyl chloride, since it generates tertiary radicals via backbiting reactions, which possess reduced reactivity and lower propagation rate constants, as previously described in kinetic and theoretical studies comparing acrylates and methacrylates.^91,92^ Furthermore, the α–methyl group induces additional steric effects. Nevertheless, surface functionalization of the **PE** film with MC was achieved.

**Scheme 1:**
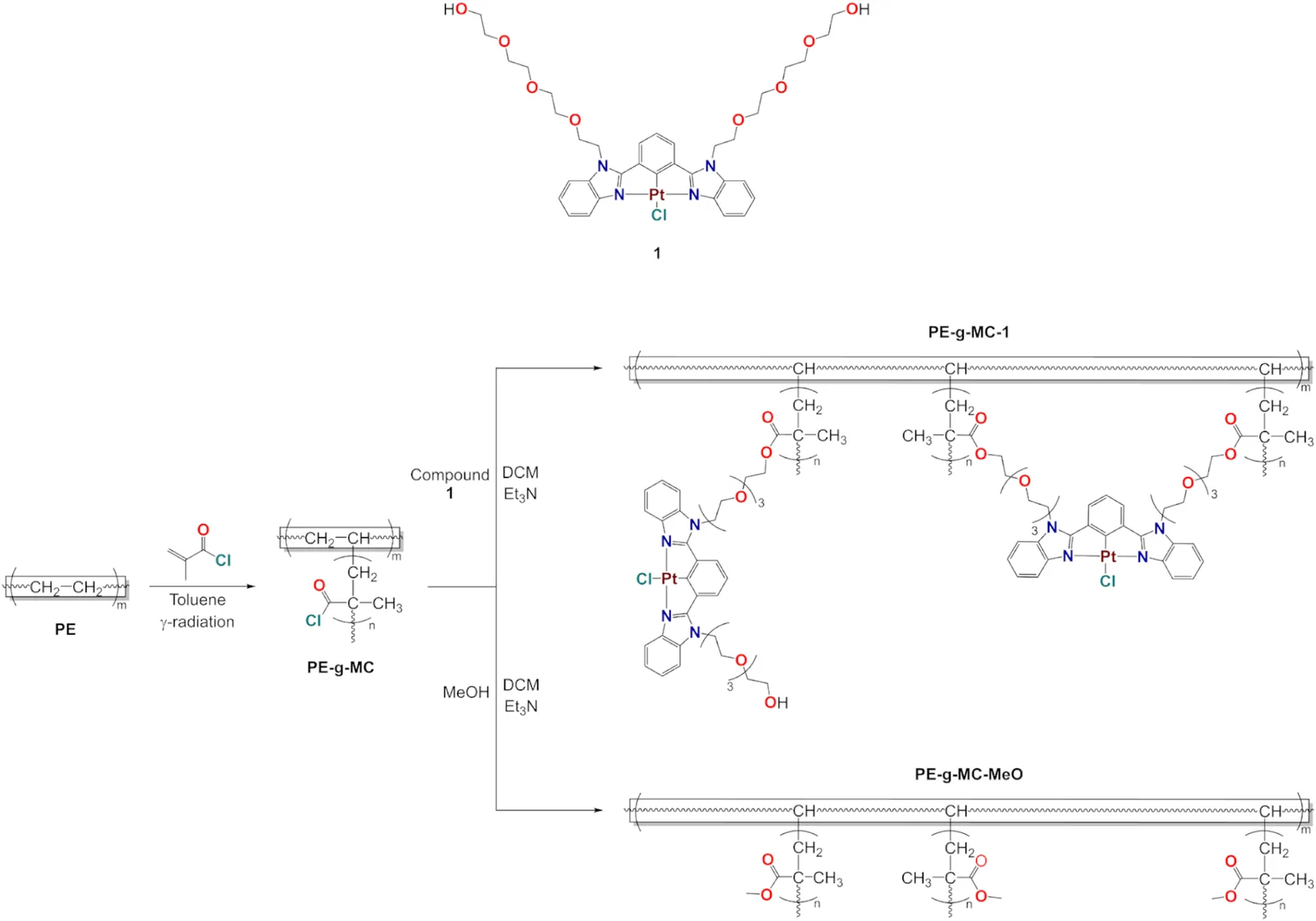
Immobilization of Pt-NCN Complex (**1**) onto **PE-g-MC** Films, and Esterification with MeOH

**Table 1:** Grafting and Anchoring Percentages (% Weight) of Films Irradiated at 2, 4, 6, and 8 kGy

Dose (kGy)	Polymer film	Incorporated group	Grafting (% weight)^a^	Anchoring_complex **1**_ (% weight)^b^
2	**PE-** ***g*** **-MC-1-2kGy**	**1**	4.27	1.36
	**PE-** ***g*** **-MC-MeO-2kGy**	MeO	3.11	NC^c^
4	**PE-** ***g*** **-MC-1-4kGy**	**1**	8.15	1.70
	**PE-** ***g*** **-MC-MeO-4kGy**	MeO	7.88	NC^c^
6	**PE-** ***g*** **-MC-1-6kGy**	**1**	11.38	2.23
	**PE-** ***g*** **-MC-MeO-6kGy**	MeO	9.14	NC^c^
8	**PE-** ***g*** **-MC-1-8kGy**	**1**	28.22	2.65
	**PE-** ***g*** **-MC-MeO-8kGy**	MeO	25.05	NC^c^

a The grafting (% weight) was calculated using eq 1.

b Anchoring_complex **1**_ (% weight) was calculated using eq 2.

c NC = Not calculated.

### 3.2 Characterization of **PE-g-MC-1** Films

#### 3.2.1 ATR-FTIR Analysis

ATR-FTIR spectra of the **PE-g** **-MC-1** films are shown in Figure 1. The strong bands at 2915 and 2847 cm^–1^ correspond to the asymmetric and symmetric stretching vibrations of methylene (−CH_2_−) groups, respectively. The bands at 1462 and 718 cm^–1^ were attributed to −CH_2_– scissoring and rocking vibrations, consistent with the characteristic FTIR bands reported for **PE**.^93,94^ Anchoring of compound **1** onto the films resulted in new bands, whose intensities augmented with the increasing irradiation dose. The band at 3395 cm^–1^ was assigned to the −OH stretching vibration, indicating that molecules of **1** may be anchored through a TEG chain. The carbonyl stretching vibration was observed at 1727 cm^–1^ and the band at 1158 cm^–1^ was associated with the −C–O–C– stretching vibration of the TEG chains.^95,96^

**Figure 1:**
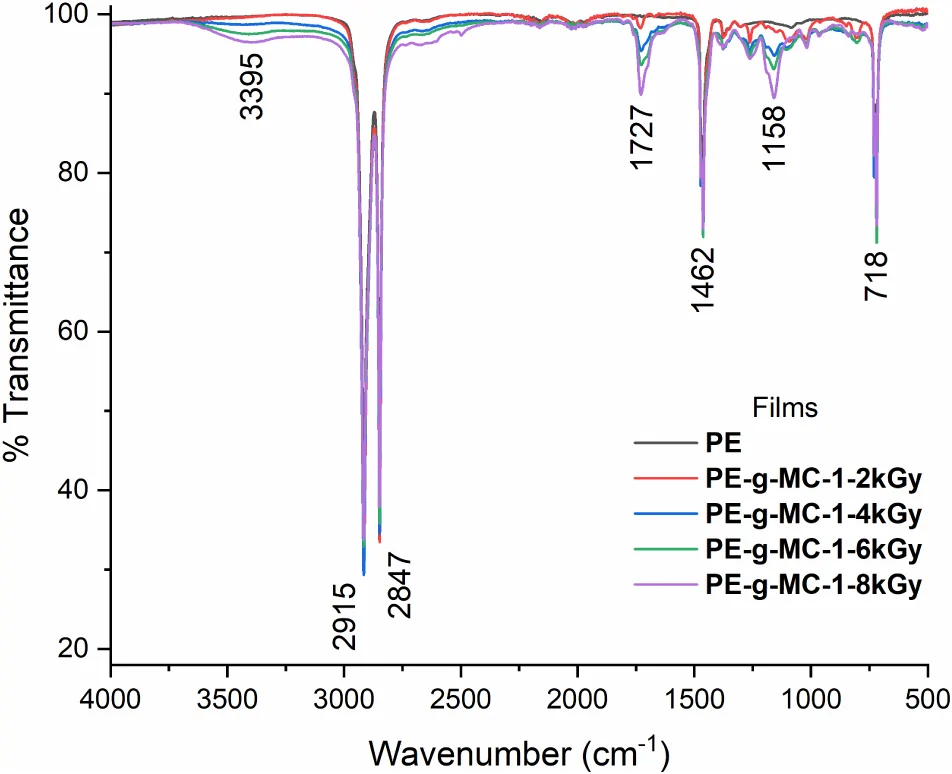
ATR-FTIR spectra of **PE-** ***g*** **-MC-1** films at different irradiation doses.

#### 3.2.2 Thermal Properties

The thermal properties of the films were studied by TGA and DSC at a heating rate of 10 °C min^–1^ under a nitrogen atmosphere. TGA and derivative thermogravimetry (DTG) curves of the **PE** film revealed good thermal stability up to 400 °C. Subsequently, the decomposition of the polyethylene backbone was observed between 420 and 500 °C (Figure 2A). Interestingly, T_5_ and T_10_ values of the films decreased with increasing anchoring of compound **1** (see Table 2), indicating that its incorporation makes the polymer more susceptible to degradation. Among the anchored films, **PE-g** **-MC-1-8kGy** exhibited the lowest thermal stability and showed two weight–loss steps at ∼150 and 250 °C (Figure 2B). These events can be attributed to the thermal degradation of the TEG chains, whose decomposition begins around 150 °C and proceeds through multiple −C–O–C– bond cleavage processes.^97^ Despite the lower thermal stability, the luminescent polymer remains thermally stable well above room temperature, supporting its use for sensing under ambient conditions. The final residue increased from 0.8 (**PE** film) to 1.5% for **PE-g** **-MC-1-8kGy**. This increase can be attributed to the formation of platinum oxide species, consistent with the anchoring percentage of compound **1** (2.65%), which corresponds to a ∼0.62% contribution. TGA and DTG curves of the rest of the anchored films are shown in Figure S5.

**Figure 2:**
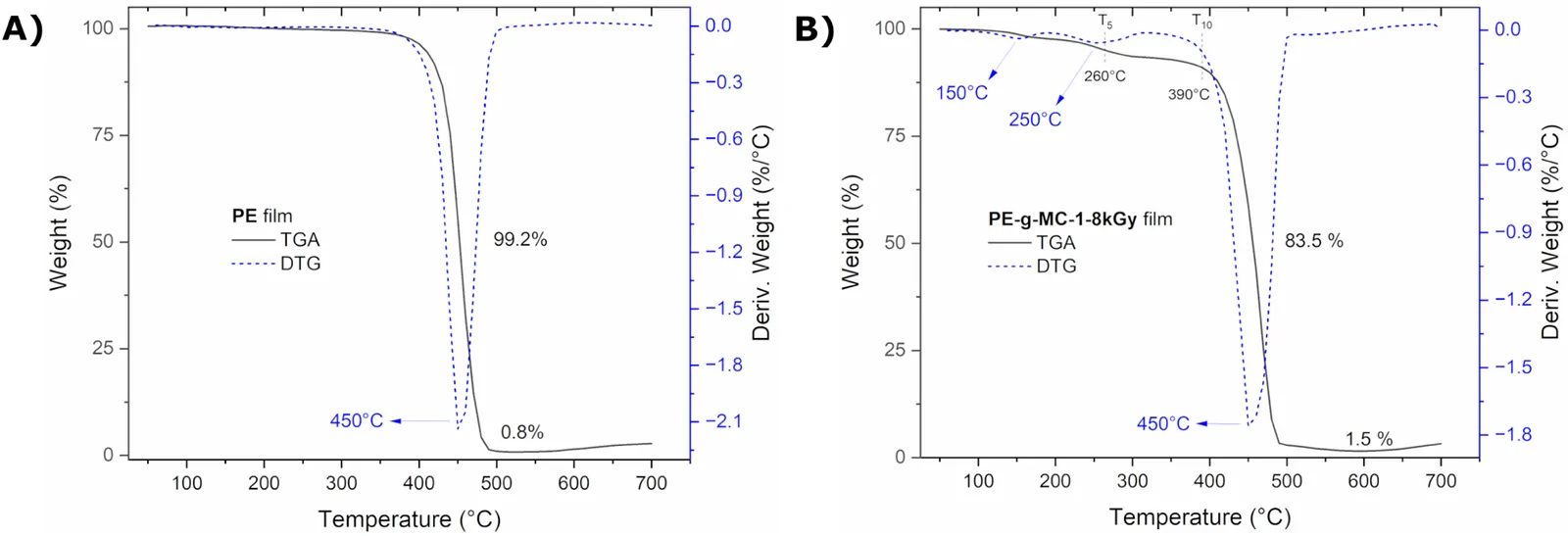
TGA and DTG curves of (**A**) **PE** and (**B**) **PE-g** **-MC-1-8kGy** films.

**Table 2:** Thermal Properties of **PE** and **PE-g-MC-1** Films

Polymer film	T_5_ (°C)	T_10_ (°C)	T_m_ (°C)
**PE**	410	420	106
**PE-** ***g*** **-MC-1-2kGy**	390	420	106
**PE-** ***g*** **-MC-1-4kGy**	400	420	106
**PE-** ***g*** **-MC-1-6kGy**	350	410	106
**PE-** ***g*** **-MC-1-8kGy**	260	390	106

According to DSC thermograms (Figure 3), anchored films exhibited an endothermic peak at 106 °C, corresponding to the melting temperature (*T*_m_) of **PE**. Although variations in the intensity of the melting endotherm were observed as a function of the irradiation dose, the melting temperature remained essentially unchanged. These changes in heat flow intensity may be associated with the combined effects of gamma irradiation, which can induce chain scission and crosslinking, and the incorporation of the grafted/anchored components into the **PE** matrix. A slight secondary endothermic event observed at 141 °C is associated with the melting of more stable polyethylene crystalline domains formed through a melting–recrystallization–remelting process, which is commonly observed in semicrystalline polyethylene.^98^

**Figure 3:**
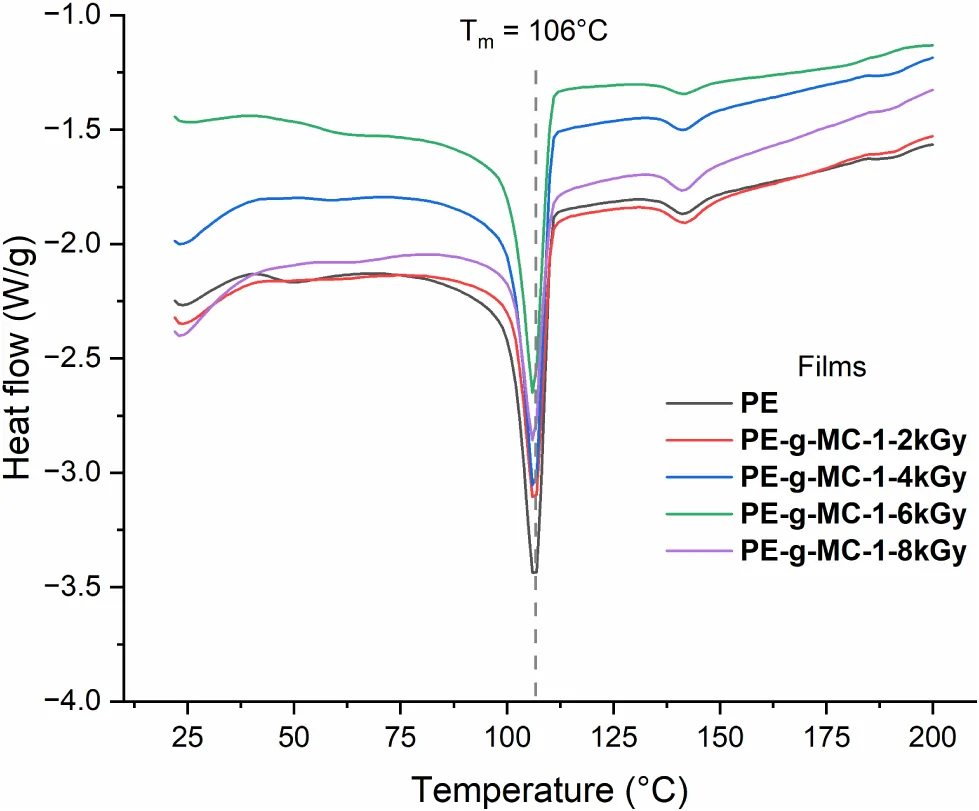
DSC curves of **PE** and **PE-** ***g*** **-MC-1** films irradiated at 2, 4, 6, and 8 kGy.

#### 3.2.3 AFM Analysis

Surface morphological changes of **PE** films after irradiation and anchoring of receptor **1** at 2 and 8 kGy were investigated by AFM analysis (Figure 4). The **PE** film exhibited Ra, Rq and Rz values of 12.1, 16.9, and 311 nm, respectively. These values indicate the presence of surface domains associated with the crystalline structure of polyethylene. After irradiation and anchoring of receptor **1**, surface roughness increased, reaching Ra, Rq and Rz values of 19.0, 24.1, and 154 nm at 8 kGy, respectively. In contrast, esterification with MeOH at 8 kGy produced an even higher roughness. This difference may be associated with the partial coverage of the grafted surface by the immobilization of complex **1**, leading to a more homogeneous surface morphology. Surface height distribution histograms further support these observations: **PE** film showed a narrow and unimodal distribution, indicating a homogeneous surface (Figure S6A), while the irradiated films (2 and 8 kGy) displayed asymmetric distributions consistent with the formation of surface protrusions (Figure S6B–D).

**Figure 4:**
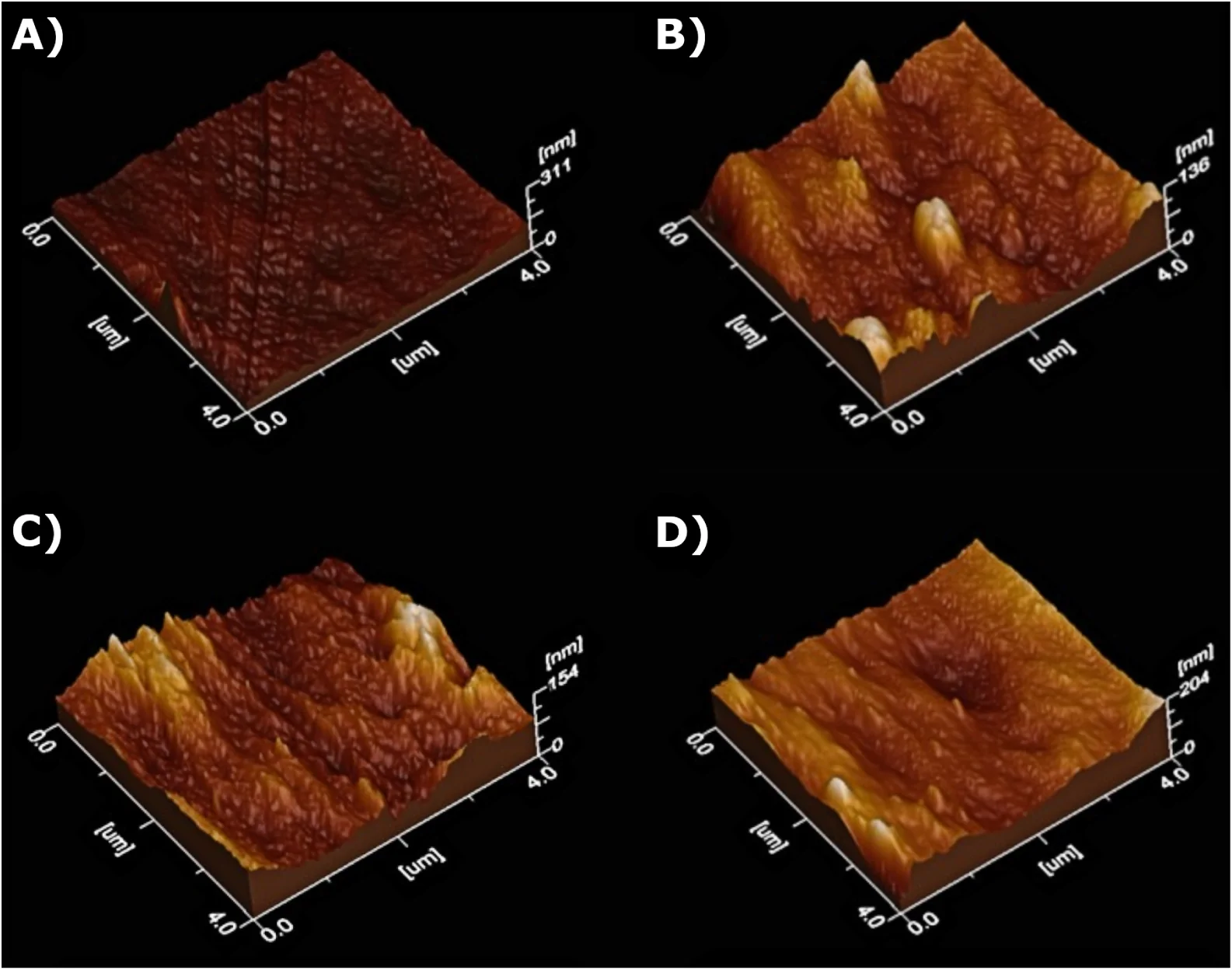
AFM topography images of (**A**) **PE**, (**B**) **PE-** ***g*** **-MC-1-2kGy**, (**C**) **PE-** ***g*** **-MC-1-8kGy** and (**D**) **PE-** ***g*** **-MC-MeO-8kGy** films.

#### 3.2.4 SEM-EDS Analysis

The elemental composition of the **PE-g** **-MC-1-8kGy** film was investigated by SEM-EDS analysis. SEM images revealed smooth regions and irregular domains, indicating a heterogeneous surface (Figure 5A). EDS analysis revealed significant differences in the atomic percentages of platinum, chlorine and oxygen in the brighter regions (Figure 5B). These variations suggest that the anchoring process occurred in a non–uniform manner, leading to domains enriched with receptor **1**. This heterogeneity may influence analyte diffusion, accessibility to active sites, and the emission response of the film samples during sensing. Overall, SEM-EDS analysis confirmed a heterogenous incorporation of receptor **1** on the functionalized polyethylene surface.

**Figure 5:**
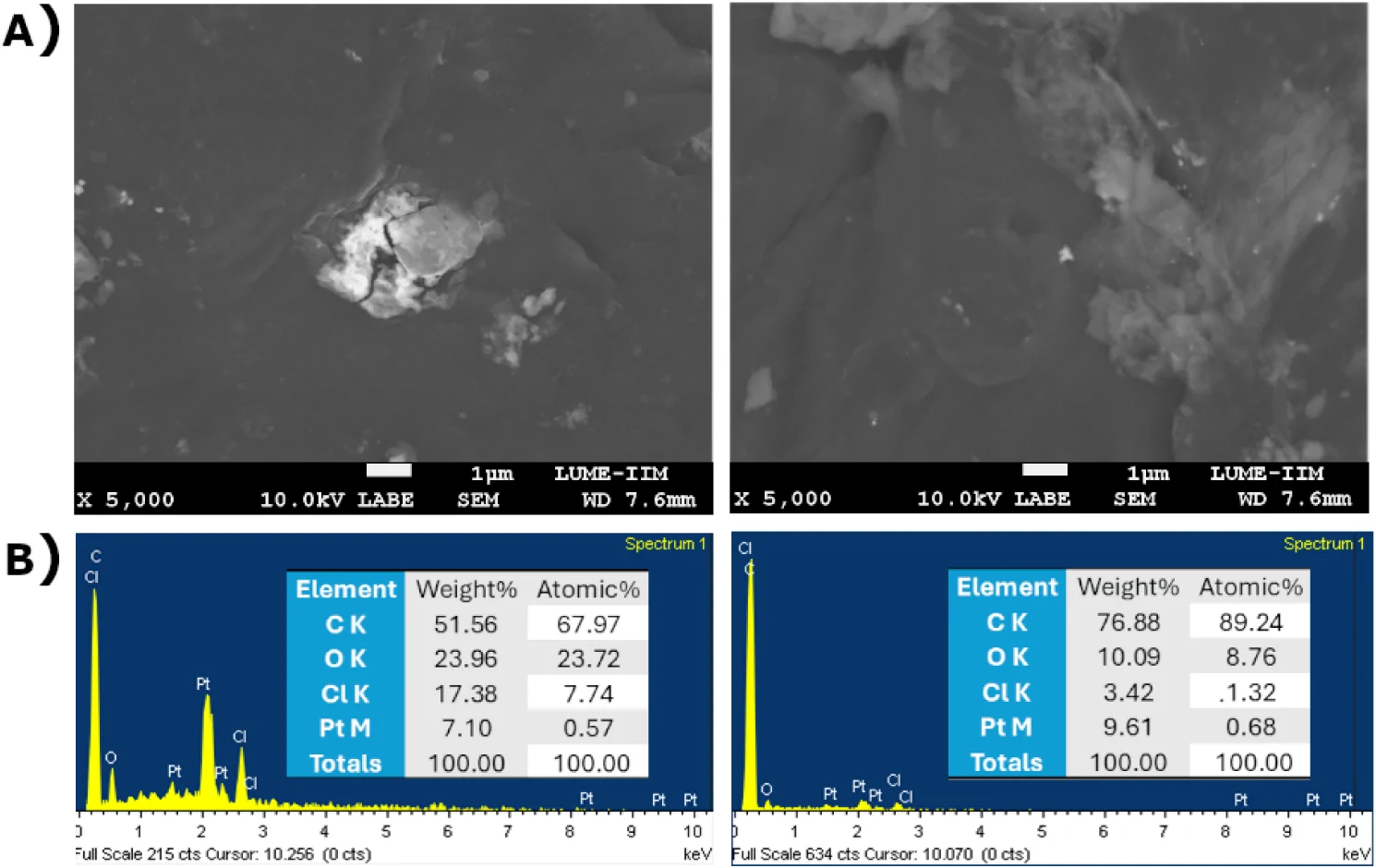
(**A**) SEM images of **PE-** ***g*** **-MC-1-8kGy** film. (**B**) Corresponding EDS spectra together with quantitative elemental analysis.

### 3.3 Optical Properties

**PE-g** **-MC-1** films were studied as circular samples (7 mm diameter) by UV-vis and fluorescence spectroscopies, and the corresponding spectra are shown in Figure 6A and B, respectively. A higher–energy absorption band was observed at 308 nm, which was assigned to the intraligand (IL) [π → π*] transition of the NCN ligand, while a lower–energy absorption band around 380 nm was ascribed to mixed IL [π → π*] and metal–to–ligand charge transfer (MLCT) [dπ­(Pt) → π*] transitions.^99,100^ Upon excitation at 380 nm, a vibronic–structured emission band was observed at 475–675 nm, originating from the ^3^IL [π → π*]/^3^MLCT [dπ­(Pt) → π*] excited states.^55,56^ These absorption and emission bands were consistent with those for receptor **1** in solution.^90^ Under UV light (365 nm), films exhibited a green emission as illustrated in Figure 6C.

**Figure 6:**
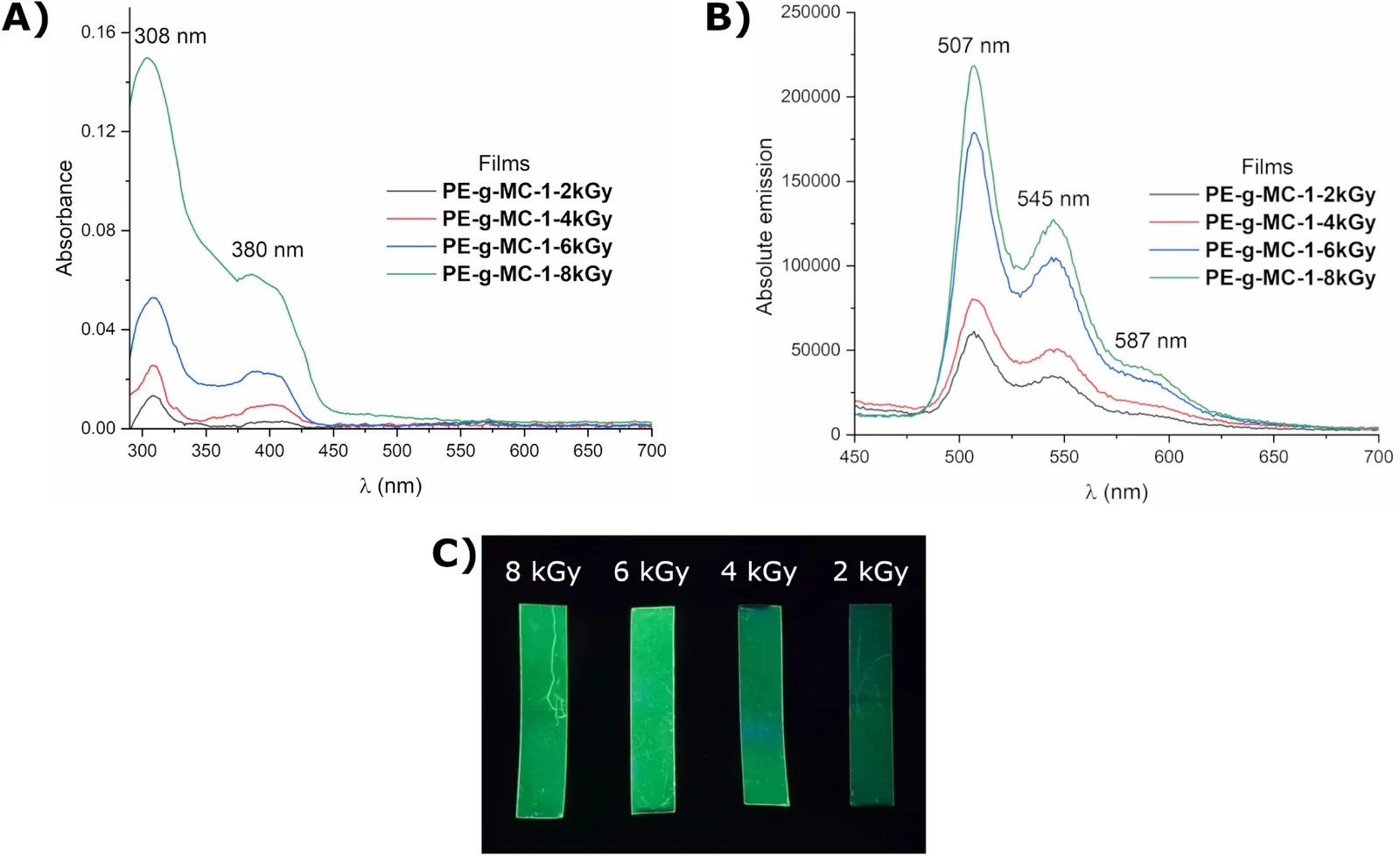
(**A**) UV-vis and (**B**) emission spectra of circular samples of **PE-g** **-MC-1** films. (**C**) Photograph of **PE-g** **-MC-1** films under UV light (365 nm).

### 3.4 Swelling Analysis

A circular **PE-g** **-MC-1-8kGy** sample was initially weighed and then evaluated by immersion in EtOH/H_2_O and DMF/H_2_O (1:1, v/v) solutions at 25 °C for 60 min. At defined time intervals, the circular sample was removed from the solution, blotted on absorbent paper, and weighed again. Swelling percentage was calculated using eq 3

3 $$\mathrm{Swelling}\;(\%)=100\left(W_{s}-W_{d}\right)/W_{d}$$

Where *W_s_* and *W*_*d*_ correspond to the weights of the swollen and dry samples, respectively.

These solvents were selected based on the solubility of receptor **1** and their miscibility with water. The 1:1 (v/v) proportion was chosen to ensure solubility of both the analyte and receptor **1**.

The swelling behavior in EtOH/H_2_O and DMF/H_2_O (1:1, v/v) solutions is shown in Figure 7. DMF/H_2_O (1:1, v/v) solution exhibited a rapid initial uptake within the first 3 min, reaching a swelling percentage of ∼8%, which remained constant up to 60 min. In contrast, EtOH/H_2_O (1:1, v/v) solution displayed a two–stage swelling process. The first stage reached a swelling value of ∼12% within 5 min, while the second stage increased to ∼25% at 30 min. After this time, the swelling percentage remained constant until 60 min. Possibly, the first stage is associated with the solvation of the receptors accessible at the surface, whereas the second stage may correspond to progressive solvation of deeper receptor units. The higher swelling observed in EtOH/H_2_O compared to DMF/H_2_O may arise from differences in solvent–polymer and solvent–solvent interactions, which can affect solvent penetration within the functionalized polyethylene matrix. Similar solvent–dependent swelling behavior has been attributed to the balance between polymer–solvent and solvent–solvent interactions in polymeric networks.^101,102^ Therefore, an EtOH/H_2_O (1:1, v/v) solution can contribute more analyte diffusion and interaction with receptor **1**, leading to an enhanced sensing response compared to a DMF/H_2_O (1:1, v/v) solution.

**Figure 7:**
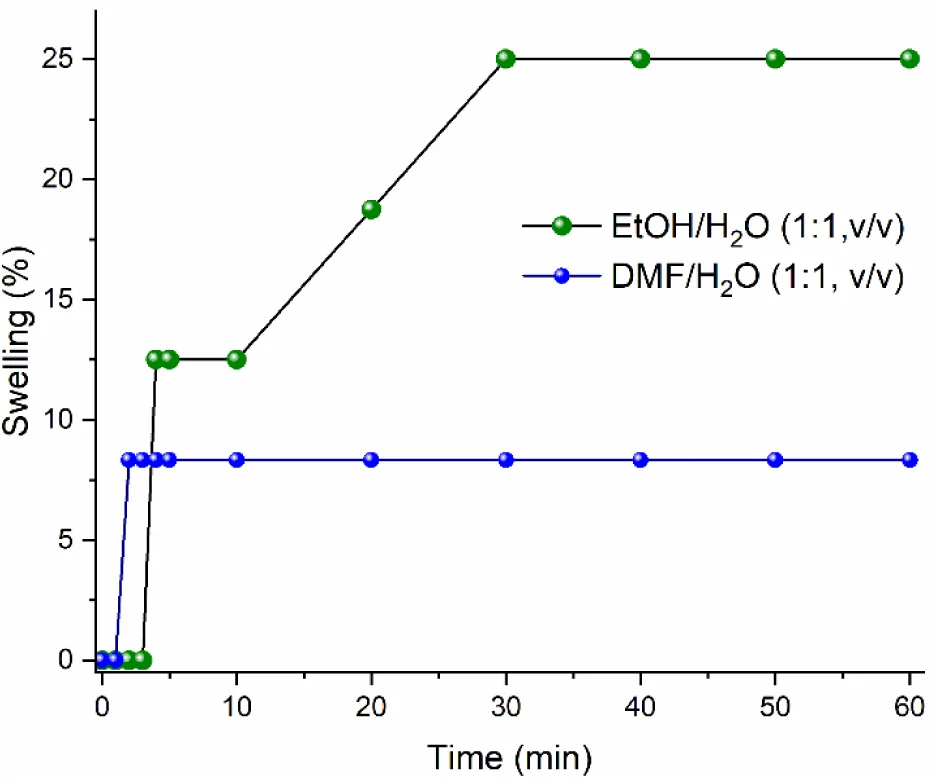
Swelling behavior of circular **PE-** ***g*** **-MC-1-8kGy** samples in EtOH/H_2_O and DMF/H_2_O (1:1, v/v) solutions.

### 3.5 Fluorometric Iodide Recognition

To evaluate the performance of **PE-g** **-MC-1-8kGy** film as a luminescent polymer chemosensor for the detection of anions, a selective experiment was performed using fluorescence spectroscopy. The experiment consisted of immersing a circular sample (7 mm diameter) in EtOH/H_2_O (1:1, v/v) solutions containing NaBr, NaI, NaSCN, NaCN, guanosine triphosphate (GTP), adenosine triphosphate (ATP), cytidine triphosphate (CTP), cysteine (l-Cys) and histidine (l-His) ([analyte] = 230 ppm) and left stirring overnight. To account for sample–to–sample variations, all sensing responses were normalized as the relative emission intensity (*I*/*I*_*o*_), where *I* and *I*_*o*_ correspond to the emission intensities after analyte exposure and in the absence of the analyte, respectively. The observed changes in the emission spectra of the luminescent polymer after the addition of analytes are shown in Figure 8A. Overall, SCN^–^, CN^–^, ATP and CTP showed a quenching effect, with emission changes below 17% (Figure 8B). GTP and H_2_PO_4_^–^ produced a moderate turn–off response, leading to an emission intensity reduction of ∼25%. In contrast, Br^–^ and l-His slightly enhanced the emission intensity (∼5%), while l-Cys caused negligible changes. Notably, I^–^ exhibited the greatest emission change, exhibiting a turn–off response with an emission intensity decrease of ∼80%. The optical response generated by Br^–^ suggests a different interaction pathway with complex **1** that does not promote the excited–state quenching observed for I^–^, despite their similar soft Lewis basicity. This distinct behavior may be associated with the higher polarizability and lower hydration energy of I^–^,^63,65^ which favors its accumulation within the polymer matrix and its interaction with the Pt-NCN unit.

**Figure 8:**
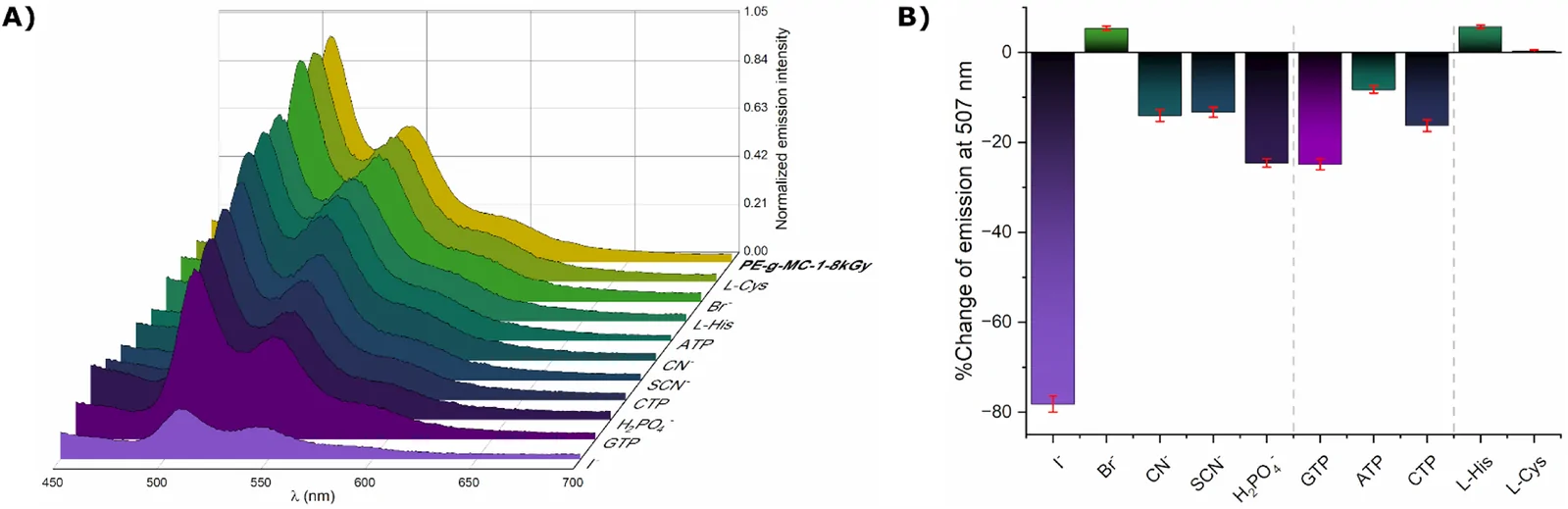
(**A**) Changes in the emission spectrum (λ_ex_ = 380 nm) of **PE-** ***g*** **-MC-1-8kGy** film after the addition of analytes in EtOH/H_2_O (1:1, v/v) solution. (**B**) Emission change percentage of **PE-** ***g*** **-MC-1-8kGy** film calculated at 507 nm upon addition of analytes.

Considering that the emission change induced by I^–^ was evaluated after overnight immersion, a time–dependent study was carried out to determine the time required to reach emission equilibrium under the same conditions. As shown in Figure 9A, the emission intensity gradually decreased over time upon exposure to iodide. A pronounced decrease in emission was observed within the first 15 min, suggesting that a significant fraction of receptor **1** is accessible for interacting with iodide. Subsequently, the turn–off response reached equilibrium at 165–180 min, with a residual green emission, as illustrated in Figure 9B. The relatively slow response can be attributed to the diffusion of iodide into the luminescent polymer. The diffusion of I^–^ within the polymer matrix contributes to the overall sensing kinetics, requiring additional time for the analyte to access recognition sites located within less accessible regions of the polymer matrix. Given that the emission intensity notably decreased within the first 15 min, a lower NaI concentration (50 ppm) was selected to determine the time required to reach equilibrium. At this lower concentration, iodide is expected to interact with the most accessible recognition sites near the polymer surface. Figure S7 shows that the system reached equilibrium after approximately 1 h at 50 ppm of NaI. Therefore, a contact time of 1 h is required at 50 ppm of NaI, whereas higher concentrations (>50 ppm) require at least 3 h to reach equilibrium. Accordingly, all sensing measurements were performed after the corresponding equilibrium times were reached. Although relatively long contact times are required for iodide to access the recognition sites, the platform may be suitable for equilibrium–based applications, particularly given its selectivity, optical response, and reusability.

**Figure 9:**
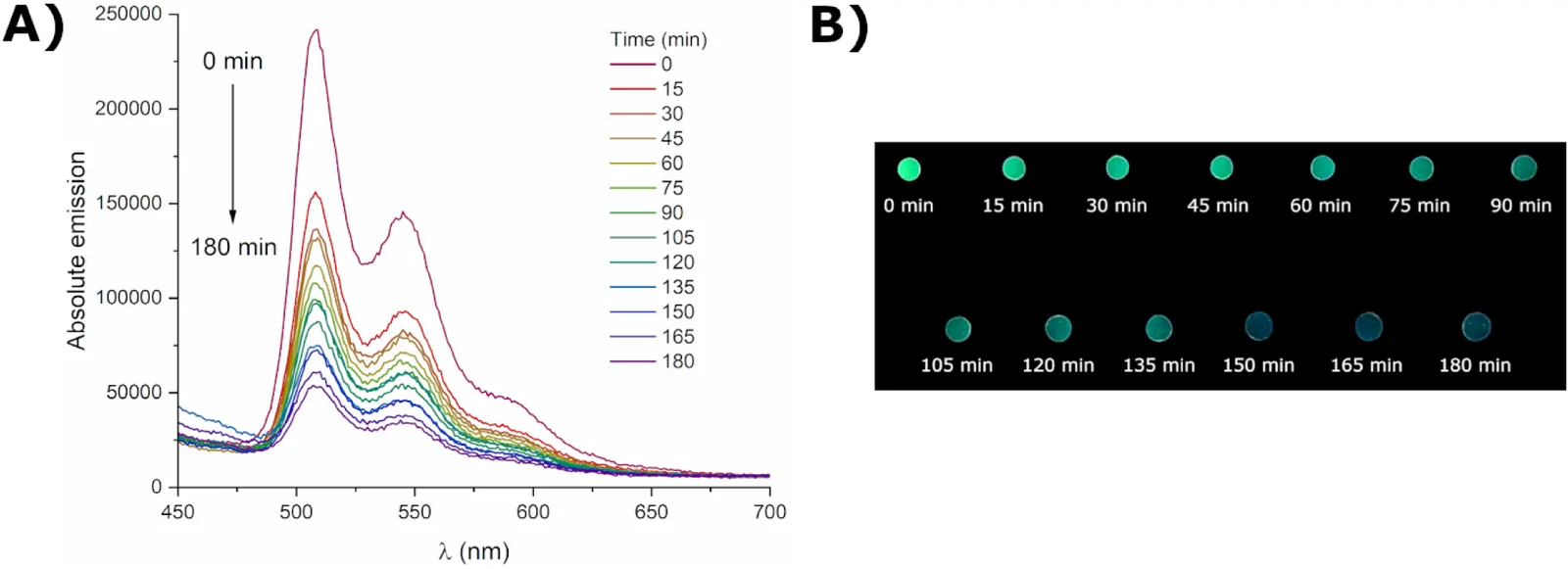
(**A**) Time–dependent emission spectra (λ_ex_ = 380 nm) of **PE-** ***g*** **-MC-1-8kGy** film upon treatment with NaI (230 ppm) in EtOH/H_2_O (1:1, v/v), recorded at 15 min intervals (0–180 min). (**B**) Photographs showing the progressive emission changes of **PE-** ***g*** **-MC-1-8kGy** film under UV light over time.

Figure 10A shows changes in the emission spectrum of the **PE-g** **-MC-1-8kGy** film upon treatment with NaI at different concentrations (0–360 ppm) in EtOH/H_2_O (1:1, v/v), after reaching equilibrium. The emission intensity dropped rapidly at low concentrations and more gradually at higher concentrations, reaching a residual green emission (Figure 10B). The quenching effect at 507 nm was analyzed using an *I*/*I*_*o*_ vs [NaI] plot, which resulted in a downward–curving Stern–Volmer response, as shown in Figure 10C. This result suggests heterogeneity in the receptor **1** population within the polymer matrix, where a fraction of receptor **1** is more accessible to quenching by iodide. This fraction can be estimated using the modified Stern–Volmer eq (eq 4), as reported for similar systems:^103,104^

4 $$I/I_{o}=\left(1-f_{a}\right)+f_{a}/\left(1+K_{q}\left[Q\right]\right)$$

where *I* and *I*_*o*_ are the emission intensities in the presence and absence of iodide, respectively; *f_a_* represents the fraction of receptor **1** accessible to I^–^, *K_q_* is the apparent quenching constant and [Q] is the concentration of NaI.

**Figure 10:**
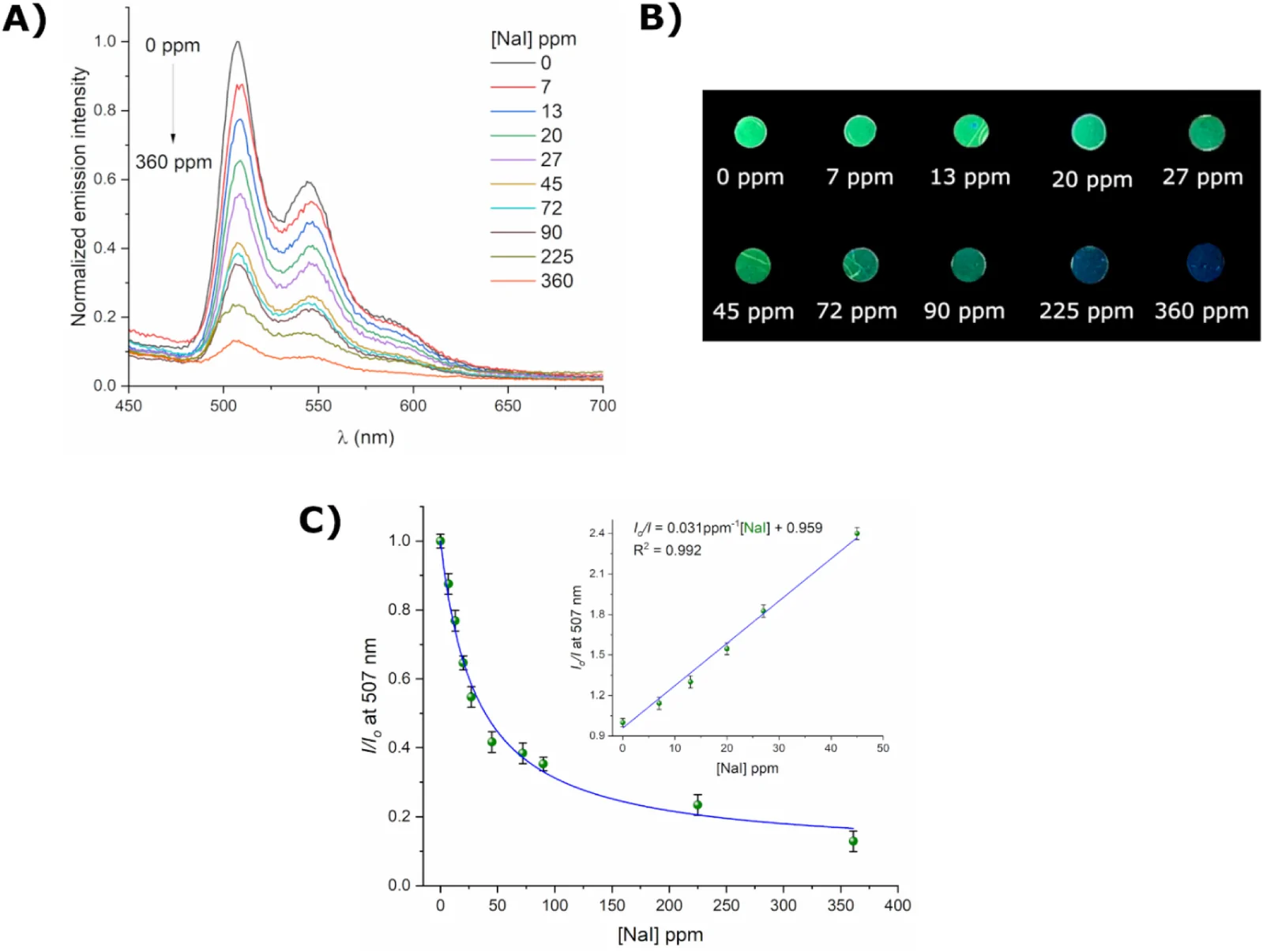
(**A**) Changes in the intensity emission (λ_ex_ = 380 nm) of **PE-** ***g*** **-MC-1-8kGy** film upon treatment with NaI at different concentrations (0–360 ppm) in EtOH/H_2_O (1:1, v/v). (**B**) Photographs show intensity emission changes at different concentrations of NaI. (**C**) Modified Stern–Volmer plot at 507 nm. The solid line represents the fit obtained using eq 4. Inset: Conventional Stern–Volmer plot at 507 nm. The solid line represents the linear fit using eq 5 over the 0–45 ppm range.

The isotherm obtained from the *I*/*I*_*o*_ vs [NaI] plot was well fitted using eq 4 (Figure 10C), yielding *f_a_* and *K_q_* values of 0.91 ± 0.03 and 4.67(±0.49) × 10^3^ M^–1^, respectively. The *f_a_* value indicates that 91% of receptor **1** is available for interaction with I^–^, while 9% is less accessible. To compare the apparent quenching constant obtained from the modified Stern–Volmer model with the classical Stern–Volmer constant (*K_SV_*), the data were also analyzed using the eq 5

5 $$I_{o}/I=1+K_{SV}\left[Q\right]$$

Accordingly, the inset of Figure 10C shows the corresponding *I*_*o*_ */I* versus [NaI] plot, for which a linear fit was obtained between 0 and 45 ppm, yielding a *K_SV_* value of 4.64(±0.20) × 10^3^ M^–1^. The similarity between both constant values indicates that the fraction of inaccessible receptor **1** has a limited impact on the quenching efficiency, suggesting that most active sites are accessible to iodide. From the linear fit, the detection limit (LOD) for I^–^ was estimated using the equation LOD = 3σ/*S* (where σ is the standard deviation of the blank and *S* is the slope of the linear fit), obtaining a value of LOD = 6.55(±0.26) ppm.

### 3.6 Emission Quenching Mechanism

The quenching of the emission **PE-g** **-MC-1-8kGy** film induced by iodide can be carried out by several mechanisms, such as the inner filter effect (IFE), Förster resonance energy transfer (FRET), the “heavy–atom” effect and photo–induced electron transfer (PET). In this case, FRET and IFE mechanisms can be excluded because the UV-vis spectrum of I^–^ exhibits negligible overlap with both the emission and excitation spectra of **PE-g** **-MC-1-8kGy** film, as illustrated in Figure S8A and B. Since the emission arises from ^3^IL/^3^MLCT states, the quenching effect of iodide cannot be simply attributed to intersystem crossing via the classical “heavy–atom” effect. Instead, iodide is likely to reduce the population of the triplet excited state by promoting nonradiative deactivation pathways. In agreement with a previous report, the addition of I^–^ has been shown to decrease the triplet state population in systems absorbing/emitting in the blue/green range through electron transfer processes.^105^ In particular, a Pt porphyrin complex displayed a quenching response toward iodide, which was attributed to an interaction of I^–^ with the platinum center upon photoexcitation, thereby facilitating the electron transfer.^51^ Thus, a similar interaction between **PE-g** **-MC-1-8kGy** film and I^–^ may contribute to the observed quenching response.

### 3.7 Interaction Mechanism

To elucidate the binding mode between I^–^ and **PE-g** **-MC-1-8kGy** film, ATR-FTIR spectra were recorded in the absence and presence of I^–^, as shown in Figure 11. The band at 3395 cm^–1^, assigned to the −OH stretching vibration, showed slightly widening, implying hydrogen bonding between I^–^ and −OH group. In contrast, a new band appeared at 1560 cm^–1^, which can be attributed to perturbations in the vibrational modes of the C=C and C=N bonds, suggesting an interaction between iodide and the Pt-NCN system. Notably, the spectral region between 1313–1095 cm^–1^ presented the most changes, indicating that iodide also interacts with TEG chains. This behavior is consistent with a previous report on poly­(ethylene oxide) oligomers, whose amphiphilic character promotes the formation of hydrophobic domains where the structure of water molecules is disordered.^69^ These water molecules can be readily displaced by weakly hydrated anions such as iodide, favoring their accumulation near the hydrophobic domains and reflecting an ion–specific effect.

**Figure 11:**
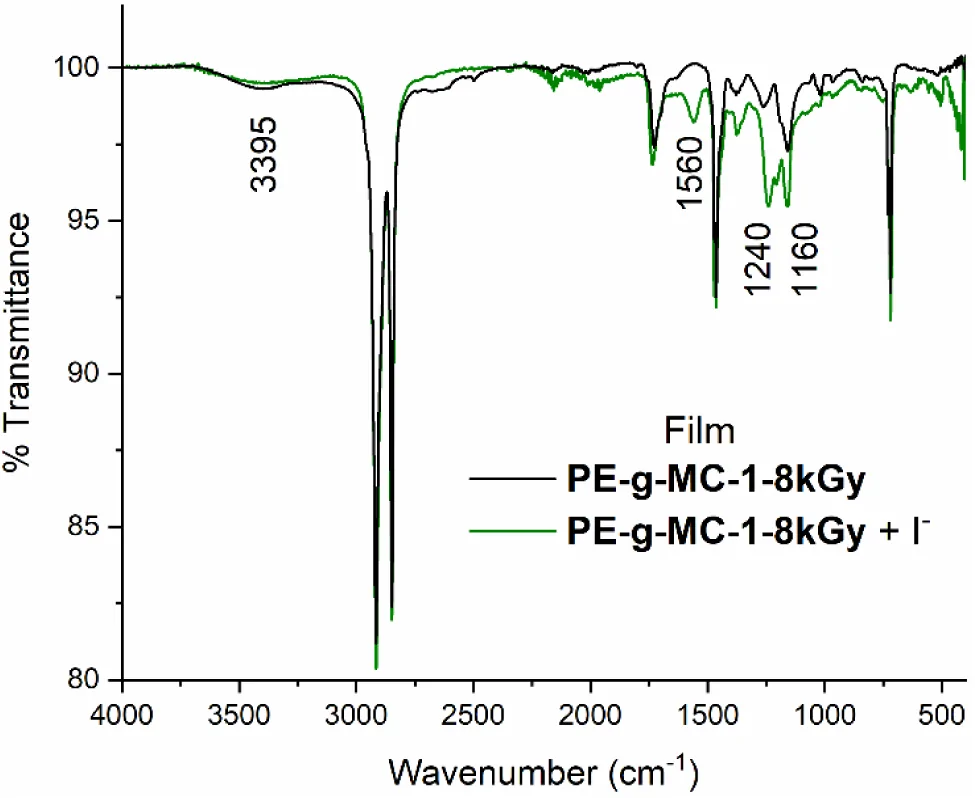
ATR-FTIR spectra of **PE-** ***g*** **-MC-1-8kGy** film in the absence and presence of I^–^.

In accordance with the changes observed in the C=C and C=N vibrational modes, the interaction of I^–^ with the Pt-NCN unit may proceed via displacement of the coordinated Cl^–^ atom at the Pt center or through an outer–sphere interaction that perturbs the electronic environment of the Pt-NCN unit without Cl^–^ substitution. Therefore, SEM-EDS analysis was performed on the luminescent polymer after treatment with 360 ppm of NaI to evaluate changes in the elemental composition associated with the presence of Cl^–^ and I^–^. SEM micrographs shown in Figure 12A revealed a heterogenous and rough surface morphology, where I^–^ was localized in the brighter regions. The atomic percentage of chlorine remained higher than that of iodine (Figure 12B), indicating that Cl^–^ remains present after iodide exposure.

**Figure 12:**
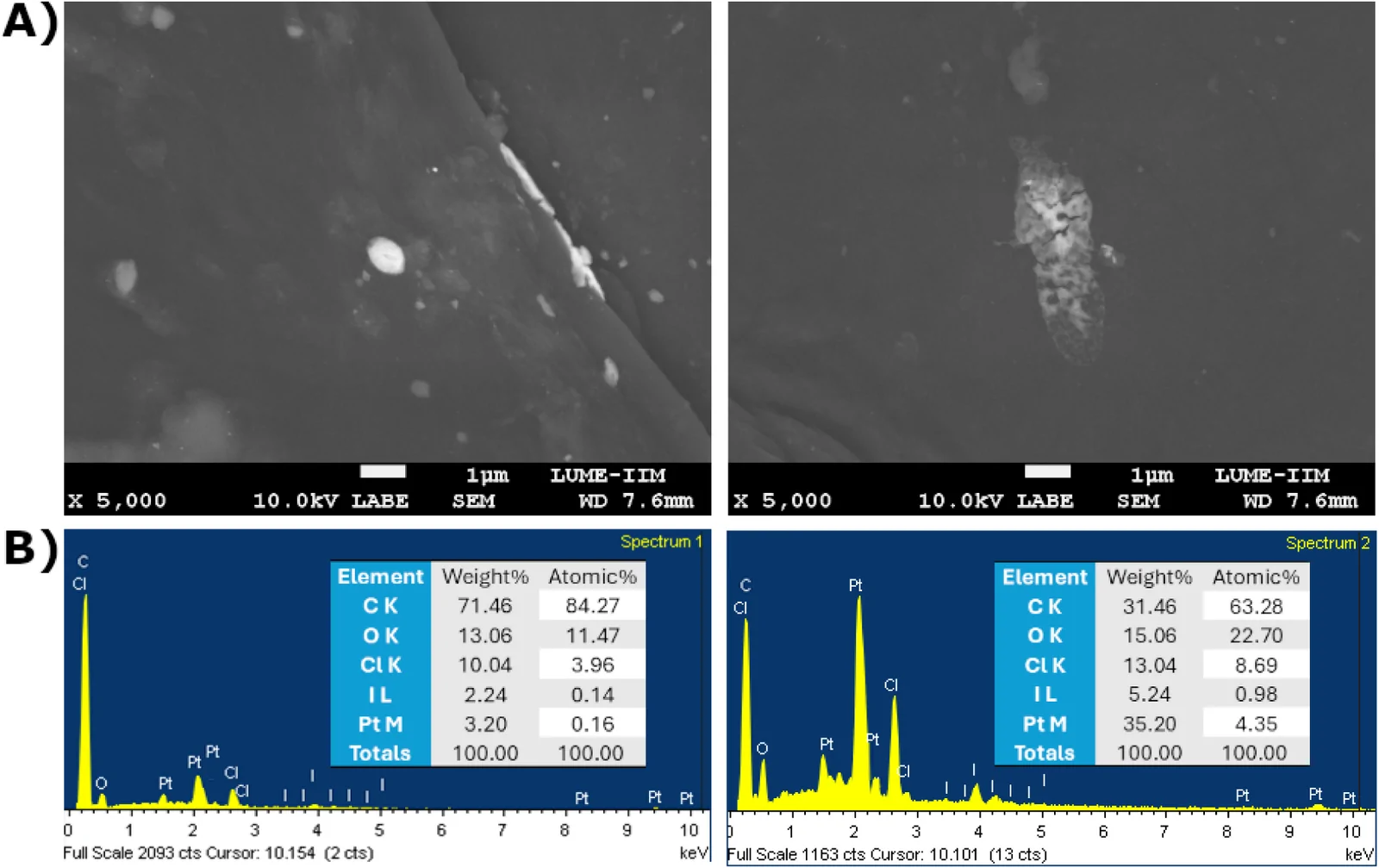
(**A**) SEM images of **PE-** ***g*** **-MC-1-8kGy** film after exposure to 360 ppm of NaI. (**B**) Corresponding EDS spectra together with quantitative elemental analysis.

Complementary XPS analysis was performed to further investigate the elemental composition and chemical environment of Pt, Cl and I. Figure 13A shows the high–resolution XPS spectrum of Pt 4f, which was deconvoluted into the spin–orbit components Pt 4f_5/2_ and Pt 4f_7/2_, centered at 75.96 and 72.64 eV, respectively. The corresponding spin–orbit splitting of 3.32 eV is consistent with the characteristic Pt 4f doublet reported (3.33 eV).^106^ Figure 13B shows the Cl 2p region, which was deconvoluted into components centered at 199.82 and 198.06 eV, assigned to Cl 2p_1/2_ and 2p_3/2_, respectively. The energy separation of 1.76 eV is consistent with the characteristic Cl 2p doublet reported.^107^ Furthermore, the binding–energy separations between Cl 2p_3/2_ and Pt 4f_5/2_ and Pt 4f_7/2_ were calculated as 122.10 and 125.42 eV, respectively. These values are comparable to those reported for Pt­(II)-Cl species.^108,109^ The deconvolution of the I 3d region was not possible due to the low signal intensity and limited signal–to–noise associated with low iodide concentration in the material, as evidenced by the XPS survey spectrum (Figure S9). Nevertheless, the persistence of the Cl 2p signal, together with the characteristic Pt 4f/Cl 2p binding–energy separations, provides additional support for the retention of a Pt–Cl coordination environment after iodide exposure.

**Figure 13:**
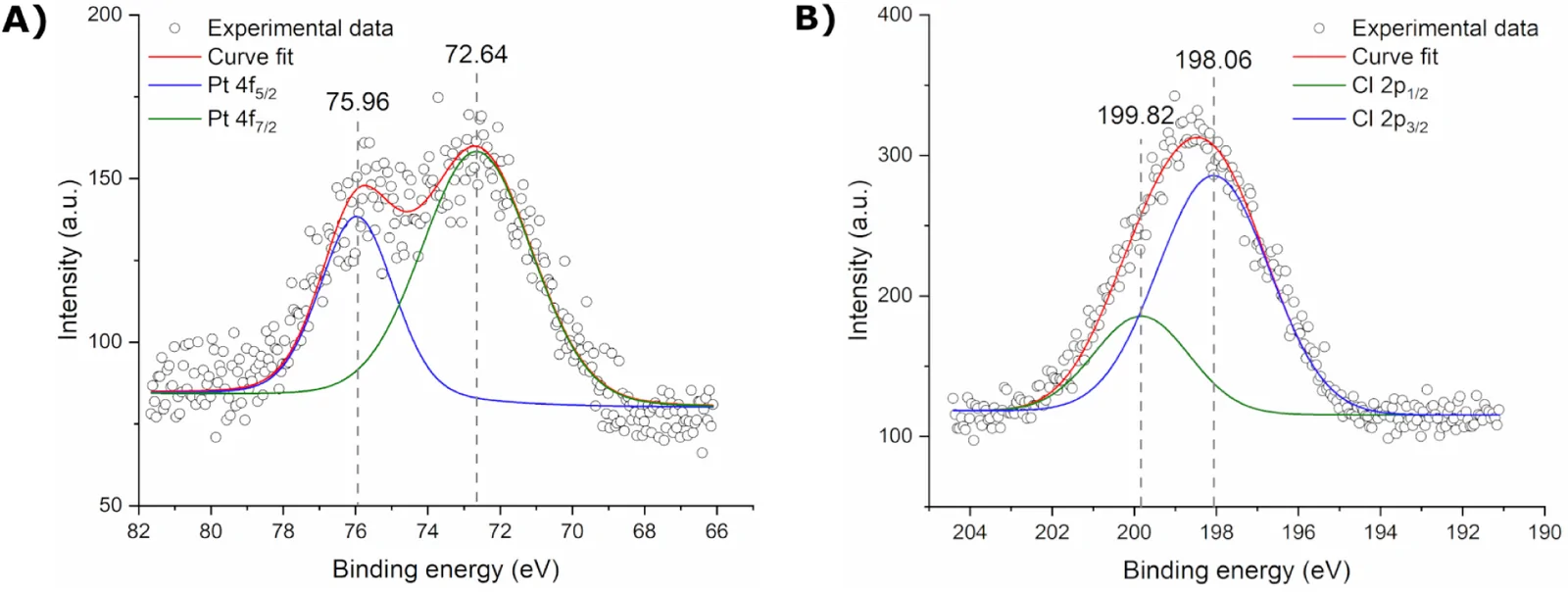
High-resolution XPS spectra of the Pt 4f (**A**) and Cl 2p (**B**) regions of **PE-** ***g*** **-MC-1-8kGy** after exposure to 360 ppm of NaI.

The C 1s and O 1s high–resolution XPS spectra were also analyzed to further support the chemical composition of the functionalized polymer. Figure S10A shows the C 1s spectrum, which is consistent with the presence of C=O, C–O, C–C, and C–Pt environments, centered at 289.05, 287.12, 284.80, and 282.92 eV, respectively. The O 1s spectrum was deconvoluted into three components centered at 533.68, 532.27, and 530.93 eV, assigned to C–O, O–C–O, and H_2_O, respectively, as illustrated in Figure S10B. XPS fitting parameters, including the binding energy, FWHM, and χ^2^ values for the fitted components, are summarized in Table S1.

Based on the ATR-FTIR, SEM-EDS, and XPS characterization, the proposed recognition mechanism involves the stabilization of I^–^ within the hydrophobic domains of TEG chains, while −OH groups may contribute through hydrogen–bonding interactions. The absence of significant changes in the Cl signal after iodide exposure, together with the XPS analysis of the Pt and Cl environments, supports the preservation of the Pt–Cl coordination sphere. Therefore, the interaction of I^–^ with the immobilized Pt-NCN complex is more attributable to an outer-sphere interaction, as illustrated in Scheme 2.

**Scheme 2:**
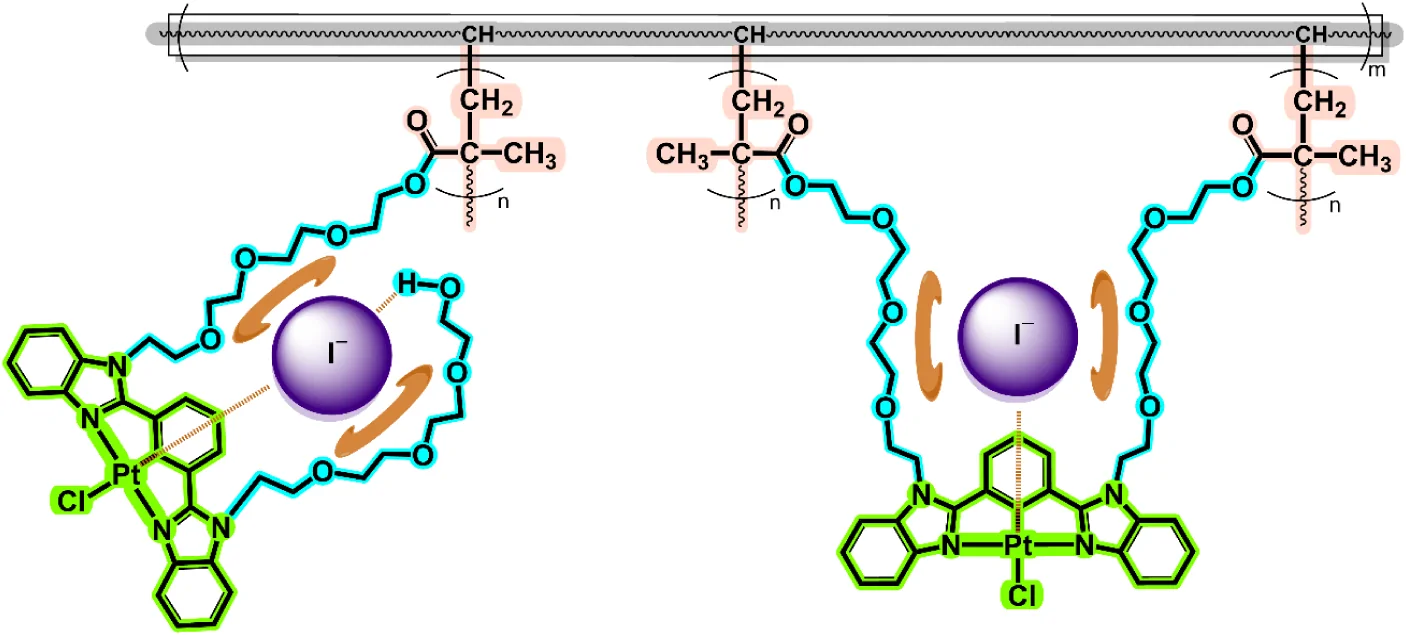
Proposed Binding Mechanism between **PE-g-MC-1-8kGy** Film and Iodide

### 3.8 Application in a Real Sample

To probe the performance of **PE-g-MC-1-8kGy** film as a luminescent chemosensor for iodide, we quantified the I^–^ concentration in a dietary supplement (DS). Figure 14 shows the changes in the emission of the luminescent polymer in triplicate measurements, all of which exhibited a turn–off response. Iodide concentration was determined using the linear fit obtained from NaI calibration, expressed in terms of I^–^ concentration (Figure 14, inset). Since the turn–off response of 50 ppm KI was similar to that observed for 45 ppm of NaI, the emission response was unaffected by the presence of the K^+^ cation (Figure S11). The KI content per tablet was determined according to the calculation procedure described in Supporting Information (pp S9 and S10). As summarized in Table 3, KI content was found from 30.1 to 34.4 mg. The mean KI content was 32.1 ± 2.1 mg (n = 3, % RSD = 6.54%), which is in good agreement with the labeled value (30 mg KI per tablet). These results demonstrated the ability of the luminescent polymer to detect iodide in a real sample.

**Figure 14:**
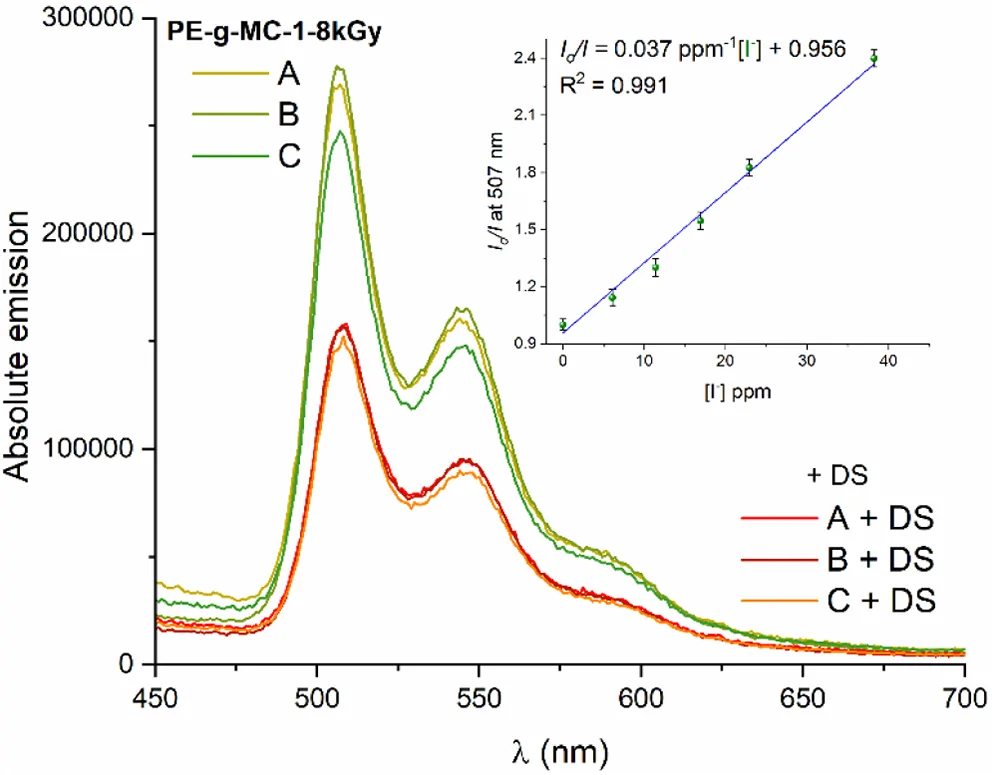
Emission intensity changes of **PE-g-MC-1-8kGy** film upon exposure to a KI–containing dietary supplement solution (n = 3). Inset: *I_o_*/*I* at 507 nm as a function of [I^–^] with the corresponding linear fit.

**Table 3:** Emission Data and KI Quantification in the Dietary Supplement Using **PE-g-MC-1-8kGy** Film

Measurement	*I*_*o*_ at 507 nm	*I*_*f*_ at 507 nm	*I*_*o*_ */I*	[I^–^] ppm	KI found (mg)
**A**	269190	155983	1.725	20.7	31.8
**B**	276270	154768	1.785	22.4	34.4
**C**	247700	147423	1.680	19.6	30.1

### 3.9 Fluorometric Mercury Recognition

A **PE-g** **-MC-1-8kGy** sample treated with 45 ppm of NaI was immersed for 1 h in solutions containing Hg^2+^, Zn^2+^, Fe^2+^, Cd^2+^, and Cu^2+^ ([analyte] = 50 ppm) in EtOH/H_2_O (1:1, v/v), using their corresponding Cl^–^ salts and emission spectra were recorded afterward. Figure S12 displays emission spectra upon the addition of metal ions, in which the emission spectrum was unchanged. Iodide is likely strongly stabilized within the hydrophobic domains of the TEG chains, limiting the accessibility of metal ions. Hence, the addition of HCl was proposed as a strategy to modify the iodide stabilization sites by partial protonation of TEG chains, increasing the hydrophilicity of the polymer and facilitating the interaction with metal ions. This approach was supported by the stability of the **PE-g** **-MC-1-8kGy** film under acidic conditions. As shown in Figure S13, no significant changes in the emission profile of the **PE-g** **-MC-1-8kGy** sample were observed after exposure to EtOH/H_2_O (1:1, v/v) containing one drop of HCl (37 wt %) for at least 3 h. In addition, the corresponding solution showed no detectable emission attributable to complex **1**, indicating that the receptor remains immobilized within the polymeric matrix. Under these acidic conditions, Hg^2+^ exhibited the greatest turn–on response (Figure 15A) of about ∼100% at 507 nm, as shown in Figure 15B. That effect is likely associated with the strong affinity of Hg^2+^ for I^–^, which may promote the formation of mercury–iodide species and restore the emission, as reported for other sensing systems.^110,111^

**Figure 15:**
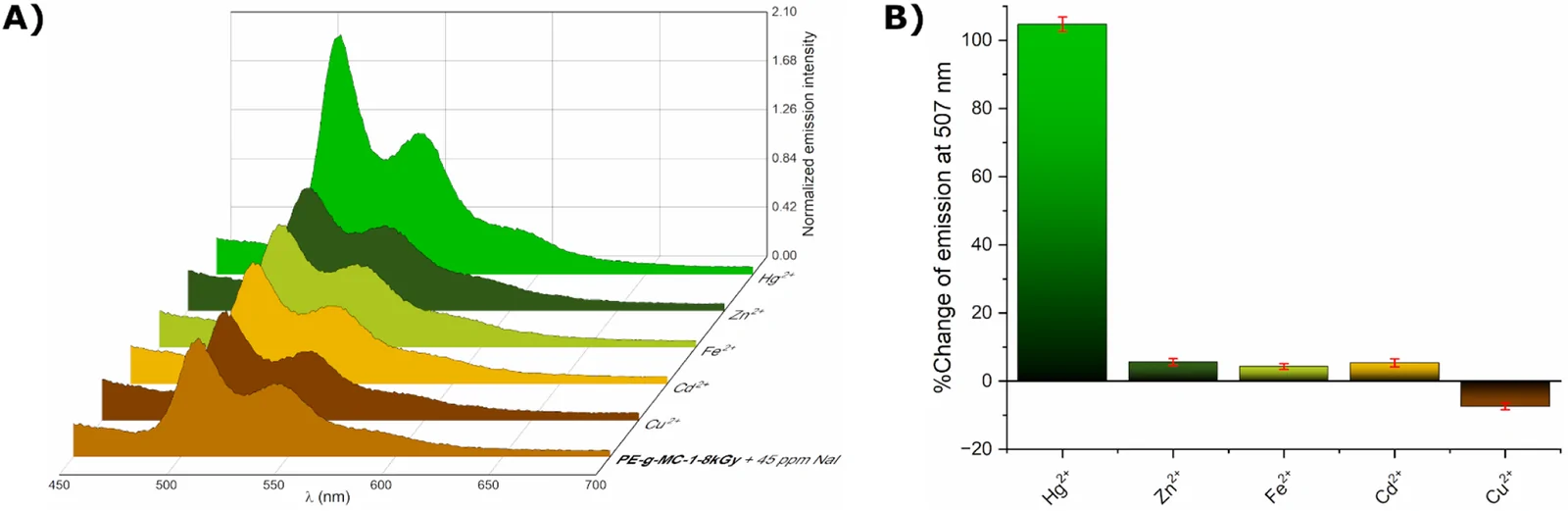
(**A**) Changes in the emission spectrum (λ_ex_ = 380 nm) of **PE-** ***g*** **-MC-1-8kGy** film treated with 45 ppm of NaI upon addition of metal ions in a EtOH/H_2_O (1:1, v/v) solution in the presence of one drop of HCl (37 wt %). (**B**) Emission change percentage of **PE-** ***g*** **-MC-1-8kGy** + 45 ppm of NaI calculated at 507 nm after addition of metal ions.

After addition of 400 ppm of Hg^2+^ and one drop of HCl (37 wt %) to **PE-g** **-MC-1-8kGy** samples treated with 225 and 360 ppm of NaI, an emission intensity increase of ∼43% was observed (Figure S14). These results indicated that the recovery of emission at high concentrations of I^–^ was limited by the stabilization of iodide within deep hydrophobic domains. The reversibility of emission was evaluated using different concentrations of HgCl_2_ (0–23 ppm) in samples treated with 45 ppm of NaI. As shown in Figure 16A, the emission intensity increased 2.5–fold in the presence of 23 ppm of Hg^2+^, reaching ∼91.7% recovery of the initial emission of **PE-g** **-MC-1-8kGy** (Figure S15). As shown in the inset of Figure 16A, the *I/I*_*o*_ ratio at 507 nm vs [HgCl_2_] plot presented good linearity (R^2^ = 0.991), with a limit of detection (LOD) value of 2.40(±0.11) ppm. The gradual recovery of the polymer emission at each Hg^2+^ concentration is illustrated in Figure 16B.

**Figure 16:**
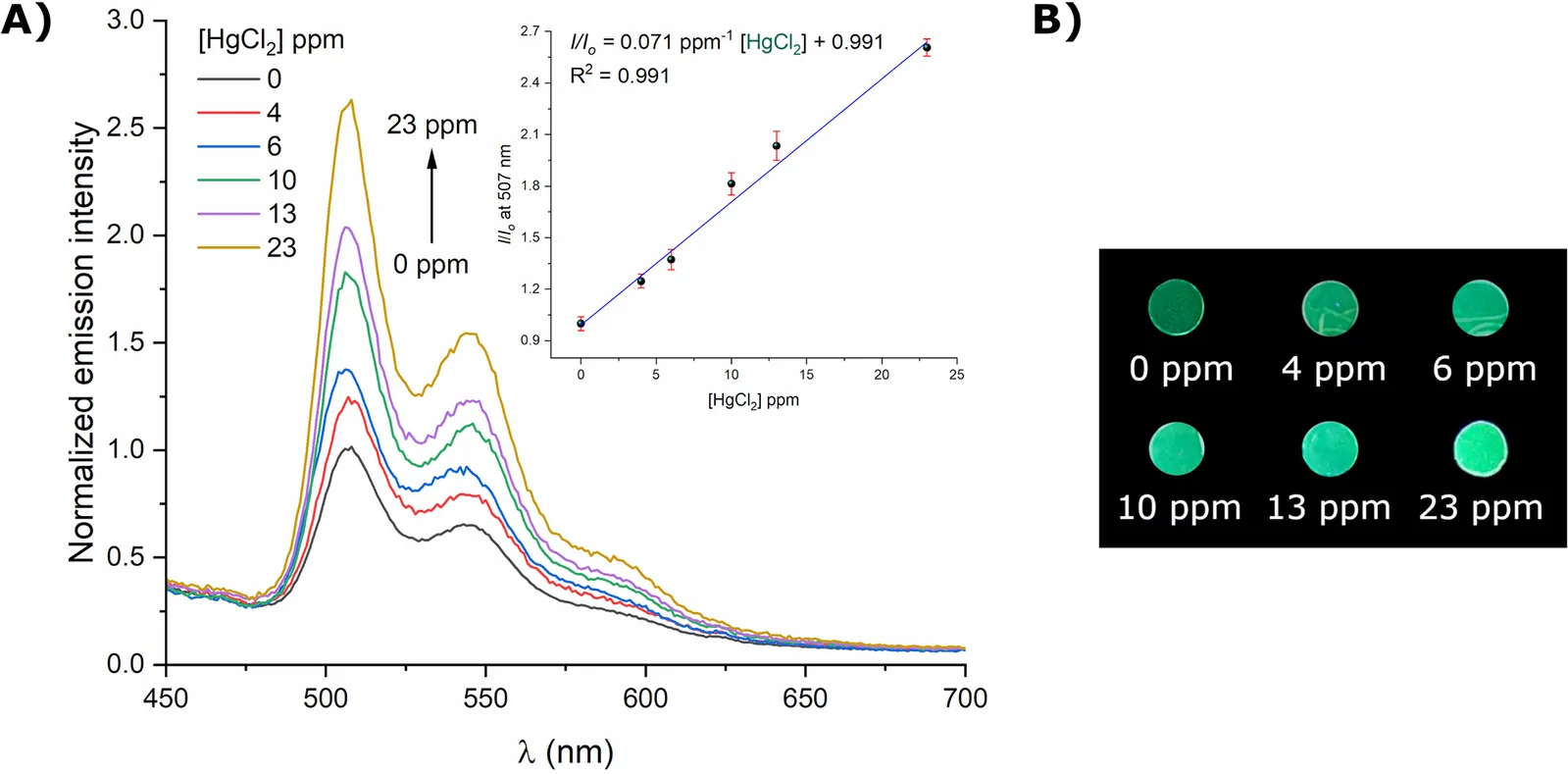
(**A**) Changes in emission intensity (λ_ex_ = 380 nm) of **PE-** ***g*** **-MC-1-8kGy** samples treated with 45 ppm of NaI upon addition of different concentrations of Hg^2+^ in EtOH/H_2_O (1:1, v/v) together with one drop of HCl (37 wt %). Inset: *I/I_o_* ratio at 507 nm vs [HgCl_2_] plot with the corresponding linear fit. (**B**) Photographs showing emission intensity enhancement of polymer films upon treatment with increasing concentrations of HgCl_2_.

The reusability of **PE-g** **-MC-1-8kGy** luminescent polymer film was investigated through alternating treatments with NaI and HgCl_2_ in the presence of one drop of concentrated HCl over several cycles. Figure 17 shows the reversible changes in the emission intensity of **PE-g** **-MC-1-8kGy** films during alternating treatment. The recovered emission intensities after the first, second and third cycles were 91.7%, 92.0% and 88.5%, respectively, relative to the initial emission intensity. The calculations of the recovery percentages are provided in the Supporting Information (pp S11). The results are presented as mean ± standard deviation (n = 3), with % RSD values ranging from 2.5% to 4.5%, demonstrating good reproducibility of the measurements (Table S2). The slight decrease in the recovered emission intensity can be attributed to partial retention of I^–^ or to gradual structural/conformational changes at the polymer surface. The recovery process was reproducible for at least three cycles, thus demonstrating the reusability of the luminescent polymer.

**Figure 17:**
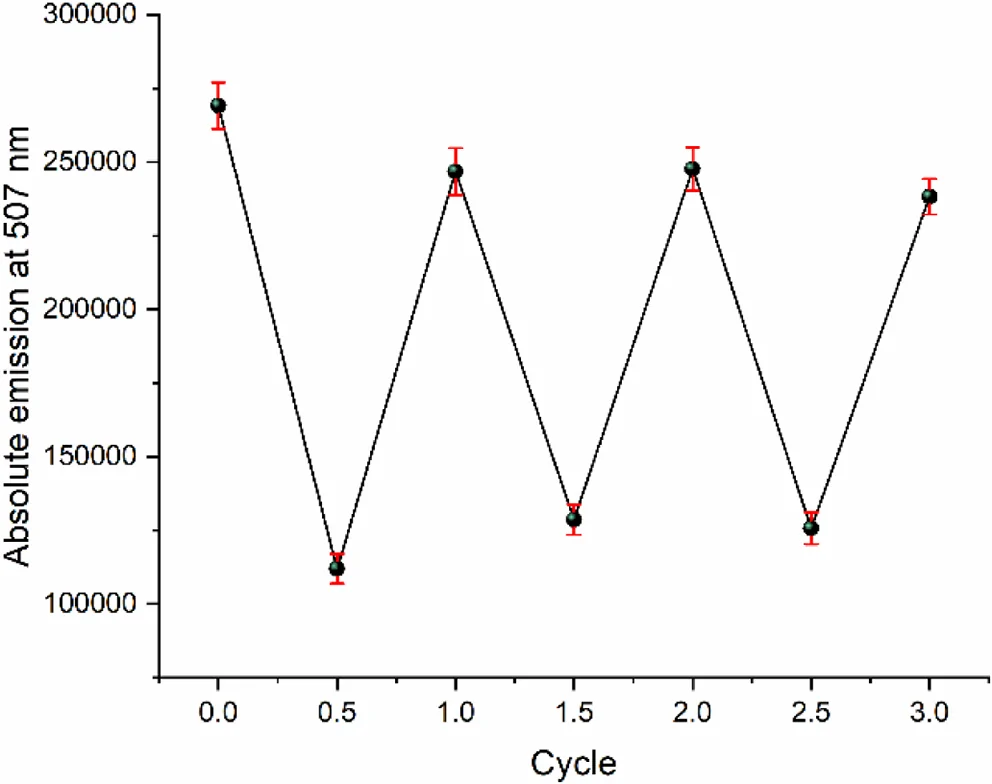
Emission intensity changes (λ_ex_ = 380 nm) of **PE-** ***g*** **-MC-1-8kGy** film over several cycles using NaI (45 ppm) and HgCl_2_ (23 ppm) in the presence of one drop of HCl (37 wt %) in EtOH/H_2_O (1:1, v/v).

A comparative table of recently reported receptors for I^–^ and Hg^2+^ are compiled in Table 4. Among these systems, several chemosensors operate in organic or organic–rich media, such as CH_3_CN or CH_3_CN/H_2_O mixtures, whereas **PE-g** **-MC-1-8kGy** enables sensing in EtOH/H_2_O (1:1, v/v). In addition, some reported receptors exhibit blue emission, whereas **PE-g** **-MC-1-8kGy** exhibits green emission, providing a distinguishable optical response. Moreover, several receptors are studied as dissolved molecular probes, which may complicate their recovery and reuse after sensing. In contrast, the immobilization of the Pt-NCN complex within the **PE** matrix provides a luminescent sensing material that can be handled and recovered, while retaining the optical response of the molecular receptor. Thus, **PE-g** **-MC-1-8kGy** combines the advantages of a luminescent receptor with those of a polymeric platform, offering an alternative strategy for the detection of I^–^ and Hg^2+^.

**Table 4:** Recent Chemosensors for the Detection and Quantification of I^–^ and Hg^2+^

Receptor	Detected ion	Sensing response	LOD	*K*_*a*_ ^a^ or *K*_*SV*_ ^b^ (M^–1^)	Medium	Reference
Fluorenone Schiff-base	I^–^	Colorimetric and Turn-on	8 nM	4.21 × 10^6^	H_2_O/THF (7:3, v/v)	^35^
PEPA-based COFs (TTETA)	I^–^	Turn-off	^c^	6.88 × 10^3^	^c^	^112^
TMB-H_2_O_2_-filter paper	I^–^	Colorimetric	0.125 μM	^c^	Aqueous media	^113^
Fluorenone-indole Schiff-base	Hg^2+^	Turn-off	^c^	9.10 × 10^3^	CH_3_CN	^114^
Imidazole-based chemosensor	Hg^2+^	Turn-on	73 nM	^c^	CH_3_CN/H_2_O (7:3, v/v)	^115^
Dithiocarbamate-OEG sensor	Hg^2+^	Turn-on	6.2 nM	^c^	Aqueous media	^116^
**PE-g-MC-1-8kGy**	I^–^/Hg^2+^	Turn-off/Turn-on	6.55/2.40 ppm	4.67 × 10^3^ ^c^	EtOH/H_2_O (1:1, v/v)	This work

a *K*_*a*_ is the apparent binding constant.

b *K*_*SV*_ is the Stern–Volmer constant.

c Data not available.

## 4 Conclusion

We have developed a luminescent polymer film as a sensor containing a Pt-NCN complex (receptor **1**) anchored to methacrylate–functionalized polyethylene films. These polymer films served as luminescent chemosensors for detection of different analytes such as anions, nucleotides, and amino acids. In the case of iodide anion, a remarkable selectivity was observed via turn–off response, with *K*_*q*_, and LOD values of 4.67(±0.49) × 10^3^ M^–1^, and 6.55(±0.26) ppm, respectively. Iodide ions were stabilized within the hydrophobic domains of TEG chains, acting as recognition sites in the luminescent polymer together with a hydrogen bond involving −OH groups. Whereas the determined LOD value for Hg^2+^ was 2.40(±0.11) ppm. As a representative application, **PE-g-MC-1-8kGy** film was employed for the determination of KI in a dietary supplement, providing results in good agreement with the labeled value. Recovery of the system emission was achieved upon treatment with Hg^2+^ in the presence of HCl. Furthermore, the luminescent polymer exhibited high emission intensity recovery over several cycles, demonstrating the reversibility and reusability of the material. In contrast to previously reported polymer–supported Pt–based systems, which have been investigated for catalytic applications, the present work explores a Pt-NCN complex anchored onto a polymer matrix functionalized by gamma irradiation, resulting in a solid–state luminescent sensing platform for the detection of iodide and mercury in solution.

## Supplementary Material



## Data Availability

The data supporting this article have been included as part of the Supplementary Information.

## Notes

^1^H, ^13^C NMR, ESI-MS spectrum, and ATR-FTIR spectrum of **1**; TGA and DSC curves of luminescent platforms; roughness parameters and surface height distribution histograms of films; spectroscopic experiments of **PE-g-MC-1-8kGy** film; calculation of KI content (mg) per tablet; reproducibility data and statistical analysis; XPS fitting parameters (PDF)
