# Supplementary material for: A Luminescent Pt-NCN Complex Anchored onto a Polyethylene Film for Sequential Detection of Iodide and Mercury
Source: ACS Omega. 2026 Sep 28;11(40):61966–83. doi: 10.1021/acsomega.6c08055 (PMC13670287; doi:10.1021/acsomega.6c08055)
Supplement: Supplementary file 1 [file ao6c08055_si_001.pdf]

# A luminescent Pt-NCN complex anchored onto a polyethylene film for sequential detection of iodide and mercury

Josue Valdes-García,[a]* Mireille Vonlanthen,[a] Fabián Cuétara-Guadarrama,[a] Ernesto Rivera,[a]* Roxana M. Calderón-Olvera,[a]* Juan Carlos Alonso-Huitrón,[a]* David Garcia-Bassoco,[a] Hector Luis Valdés-Negrín,[a] Guillermina Burillo,[b] and Alejandro Dorazco-González[c]

[a]Instituto de Investigaciones en Materiales, Universidad Nacional Autónoma de México. Circuito Exterior, Ciudad Universitaria, C.P. 04510, CDMX, México

[b]Instituto de Ciencias Nucleares, Universidad Nacional Autónoma de México. Circuito Exterior, Ciudad Universitaria, C.P. 04510, CDMX, México

[c]Instituto de Química, Universidad Nacional Autónoma de México. Circuito Exterior, Ciudad Universitaria, C.P. 04510, CDMX, México

Corresponding author: J.V.G.: valdesjosue94@comunidad.unam.mx, E.R.: riverage@unam.mx, R.M.C.O.: rcalderon@materiales.unam.mx and J.C.A.H.: alonso@unam.mx

## Supporting Information

**Figure S1**. $^1$H NMR (300 MHz, 298 K, $CDCl_3$) spectrum of **1**.
**Figure S2**. $^{13}$C NMR (75.5 MHz, 298 K, $CDCl_3$) spectrum of **1**.
**Figure S3**. Positive scan ESI-MS spectrum of **1** in acetonitrile.
**Figure S4**. ATR-FTIR spectrum of **1**.
**Figure S5**. TGA and DTG curves of anchored films at irradiated at (**A**) 2, (**B**) 4 and (**C**) 6 kGy.
**Figure S6**. Roughness parameters and surface height distribution histograms of (**A**) **PE**, (**B**) **PE-g-MC-1-2kGy**, (**C**) **PE-g-MC-1-8kGy** and (**D**) **PE-g-MC-MeO-8kGy** films.
**Figure S7**. Time-dependent emission spectra ($\lambda_{ex}$ = 380 nm) of **PE-g-MC-1-8kGy** film upon treatment with NaI (50 ppm) in EtOH/$H_2O$ (1:1, v/v), recorded at 15 min intervals (0 – 75 min).
**Figure S8**. Spectral overlap analysis between the absorption of $I^-$ and (**A**) emission and (**B**) excitation spectra of **PE-g-MC-MeO-8kGy** film.
**Figure S9**. XPS survey spectrum of **PE-g-MC-1-8kGy** film upon treatment with NaI (360 ppm) in EtOH/$H_2O$ (1:1, v/v).
**Figure S10.** High-resolution XPS spectra of the C 1s (**A**) and O 1s (**B**) regions of **PE-g-MC-1-8kGy** after exposure to NaI (360 ppm).
**Figure S11**. Changes in the emission spectra ($\lambda_{ex}$ = 380 nm) of **PE-g-MC-1-8kGy** film upon treatment with NaI (45 ppm) and KI (50 ppm) in EtOH/$H_2O$ (1:1, v/v).
**Figure S12**. Changes in the emission spectrum ($\lambda_{ex}$ = 380 nm) of **PE-g-MC-1-8kGy** film treated with NaI (45 ppm) upon addition of metal ions in EtOH/$H_2O$ (1:1, v/v) solution.
**Figure S13**. Stability of the **PE-g-MC-1-8kGy** film ($\lambda_{ex}$ = 380 nm) in EtOH/$H_2O$ (1:1, v/v) containing one drop of HCl (37 wt%) after 0, 1, 2, and 3 h. Inset: Emission spectra of the corresponding solutions collected after each exposure time.
**Figure S14**. Changes in the emission spectrum ($\lambda_{ex}$ = 380 nm) of **PE-g-MC-1-8kGy** film during sequential treatments with (**A**) 225 and (**B**) 360 ppm NaI, followed by treatment with 400 ppm $Hg^{2+}$ and addition of one drop of HCl (37 wt%) in EtOH/$H_2O$ (1:1, v/v) solution.
**Figure S15**. Sequential changes in the emission spectrum ($\lambda_{ex}$ = 380 nm) of **PE-g-MC-1-8kGy** film upon addition of 45 ppm NaI and 23 ppm $HgCl_2$, together with one drop of HCl (37 wt%), in EtOH/$H_2O$ (1:1, v/v) solution.
**Calculation of KI content (mg) per tablet**
**Reproducibility data and statistical analysis**
**Table S1.** XPS fitting parameters for the Pt 4f, Cl 2p, C 1s, and O 1s regions of **PE-g-MC-1-8kGy**.
**Table S2.** Emission recovery (%) values and relative standard deviation (RSD) during the reusability cycles of **PE-g-MC-1-8kGy.**
**REFERENCES**

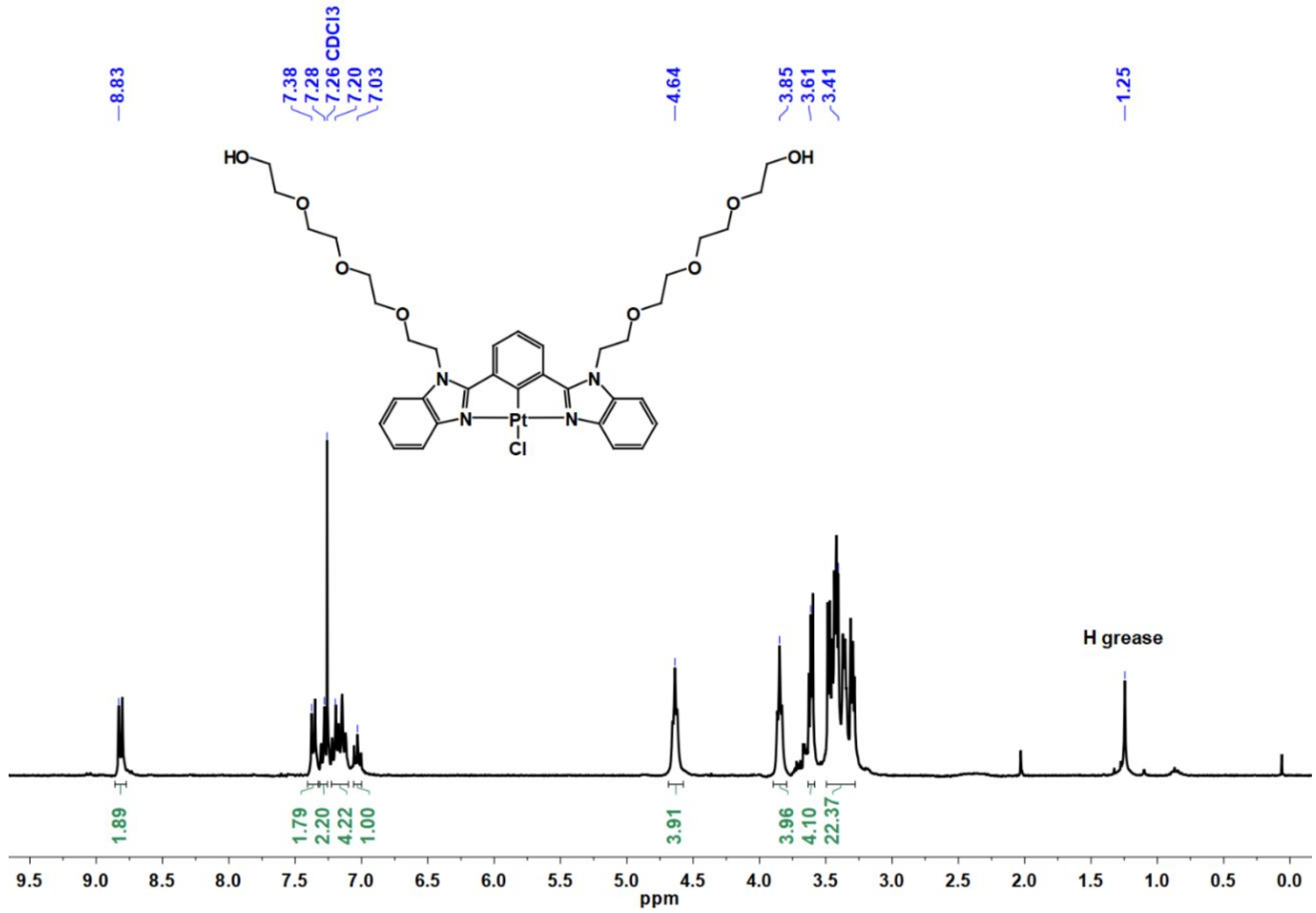


**Figure S1**. $^{1}H$ NMR (300 MHz, 298 K, $CDCl_3$) spectrum of **1**.

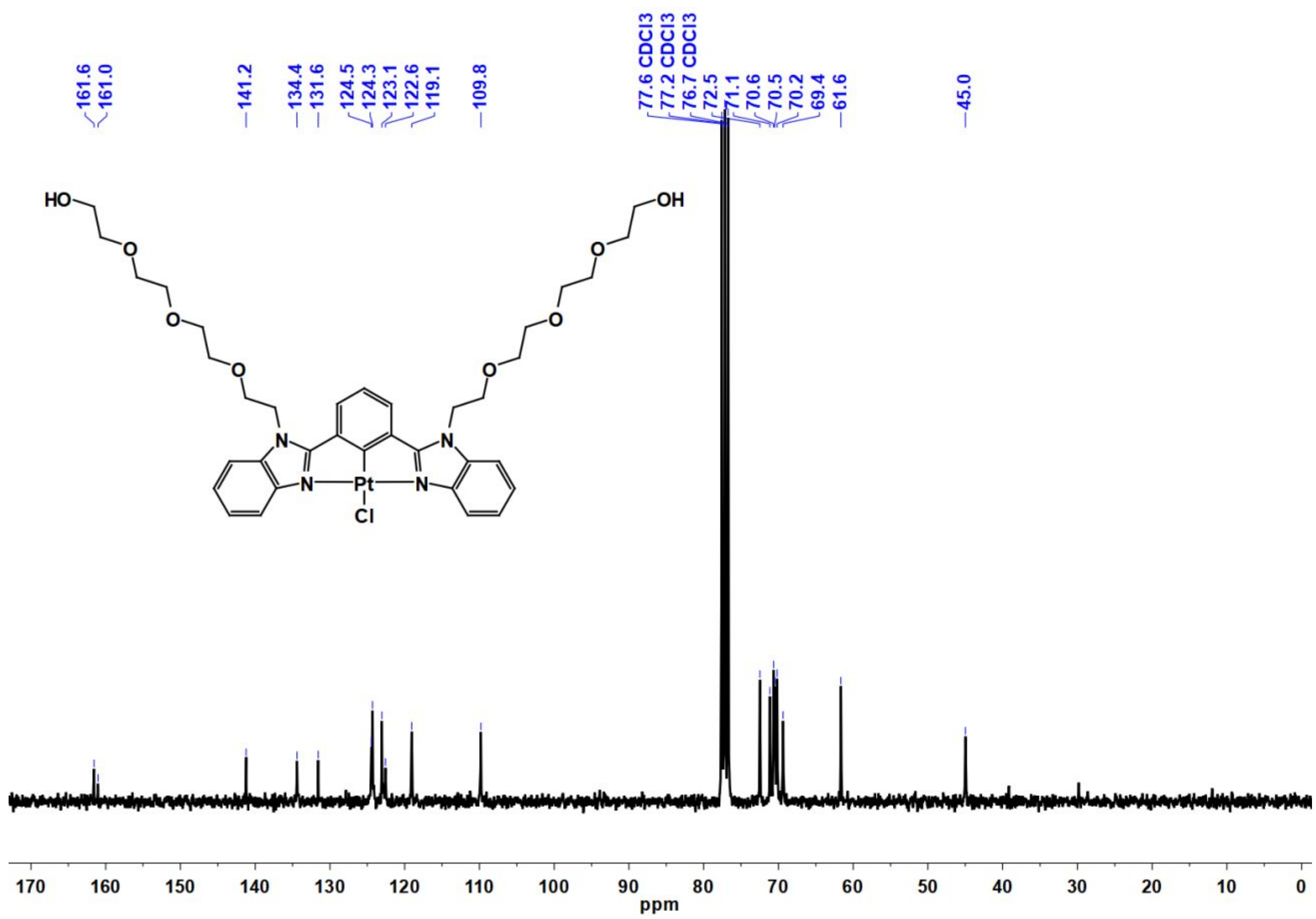


**Figure S2**. $^{13}C$ NMR (75.5 MHz, 298 K, $CDCl_3$) spectrum of **1**.

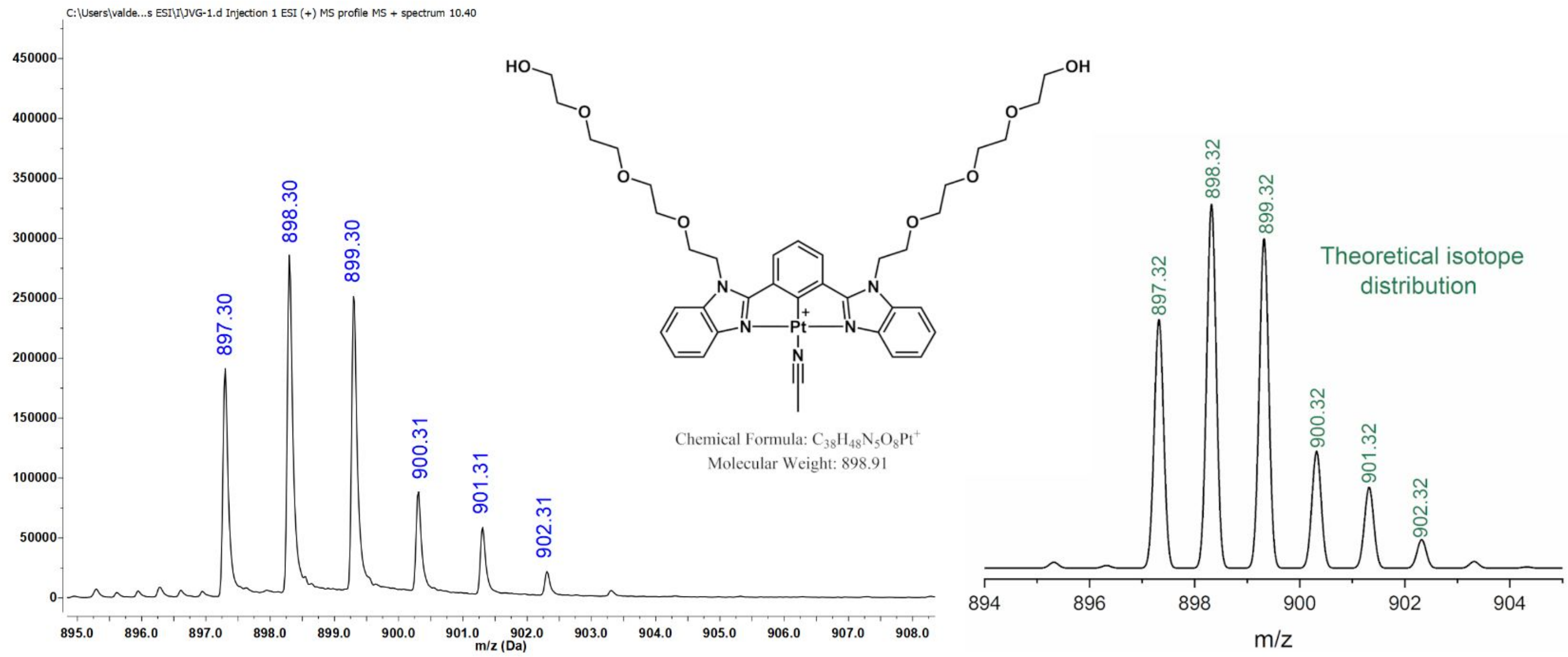


**Figure S3**. Positive scan ESI-MS spectrum of **1** in acetonitrile.

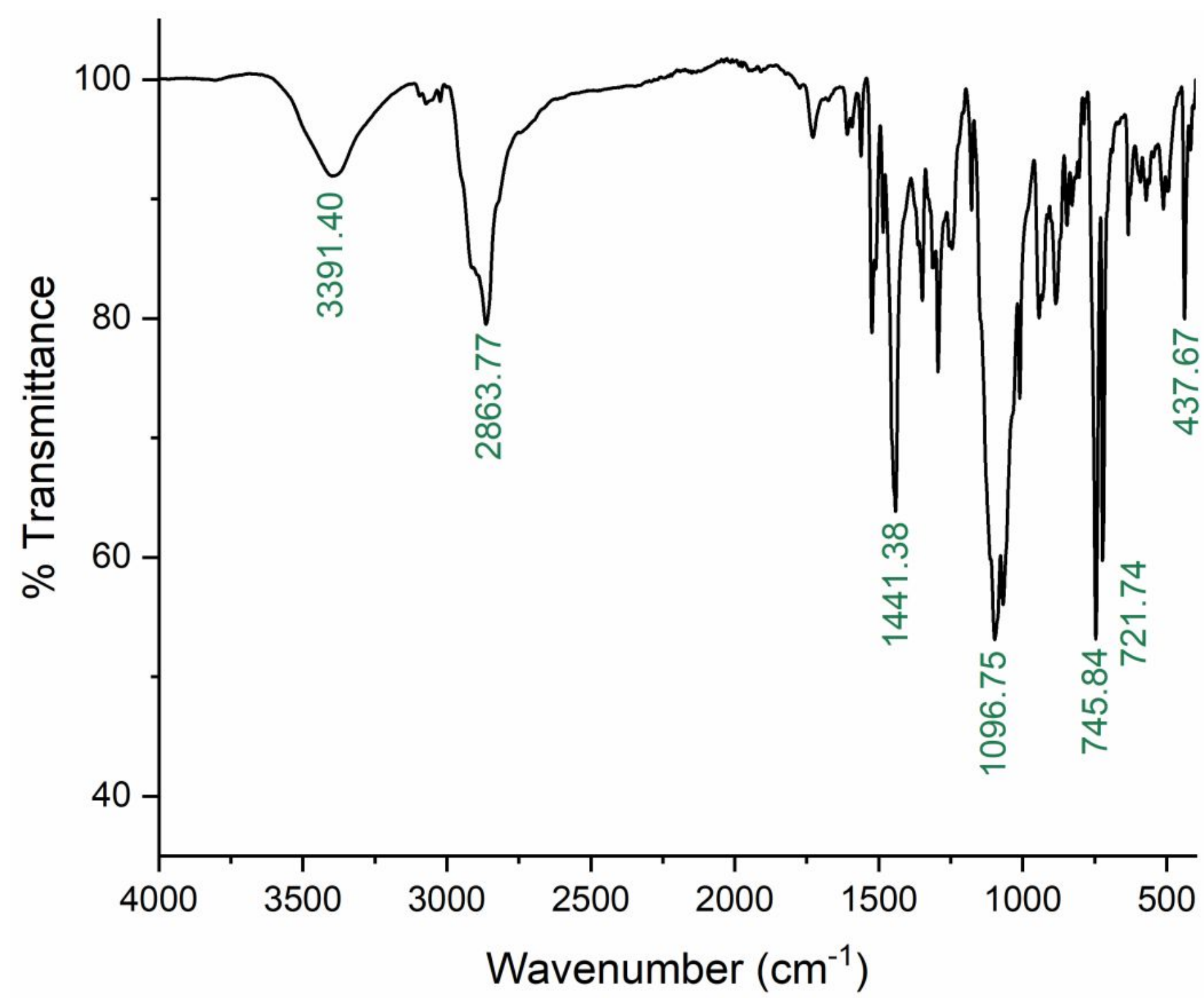


**Figure S4**. ATR-FTIR spectrum of **1**.

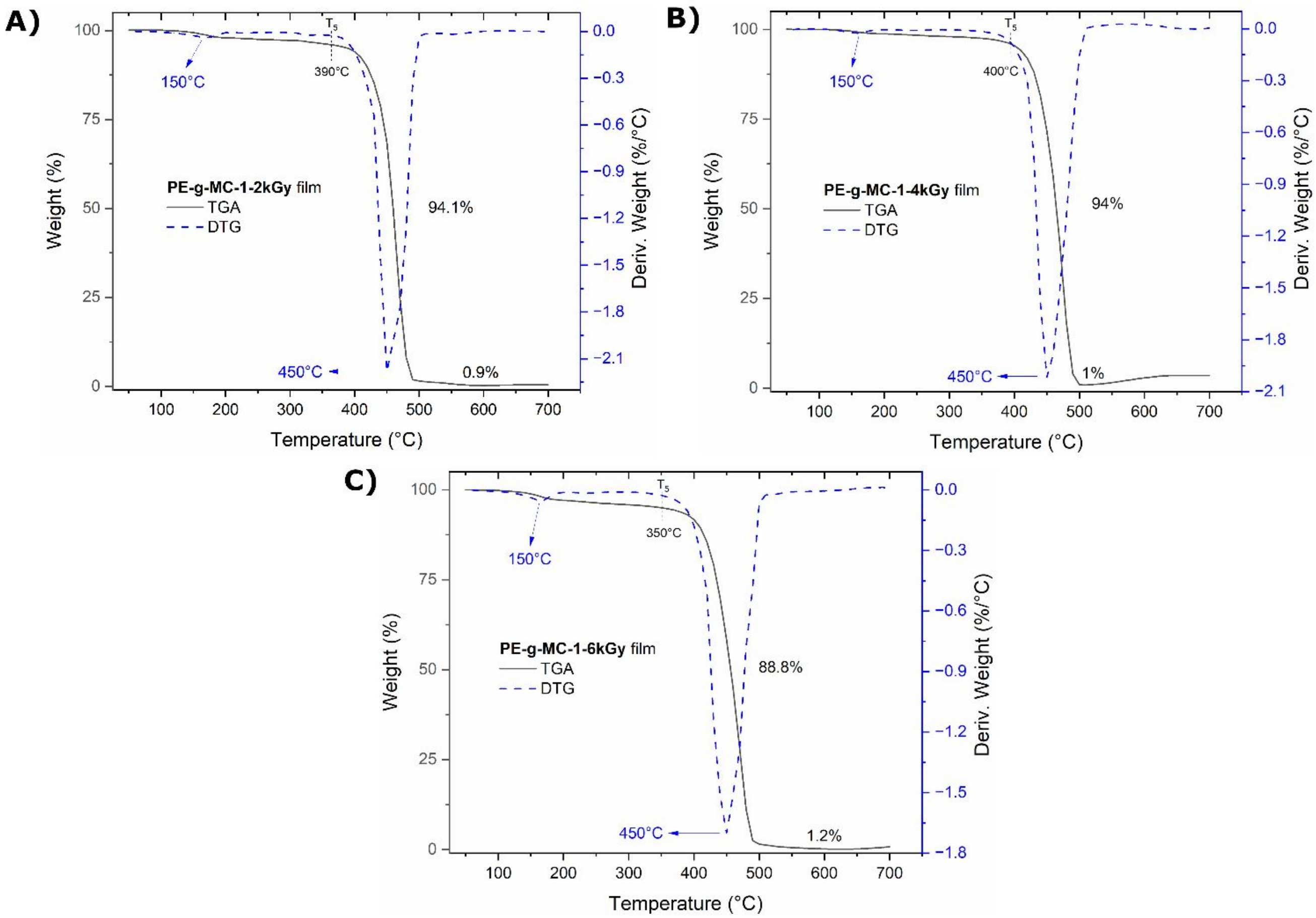


**Figure S5**. TGA and DTG curves of anchored films at irradiated at (**A**) 2, (**B**) 4 and (**C**) 6 kGy.

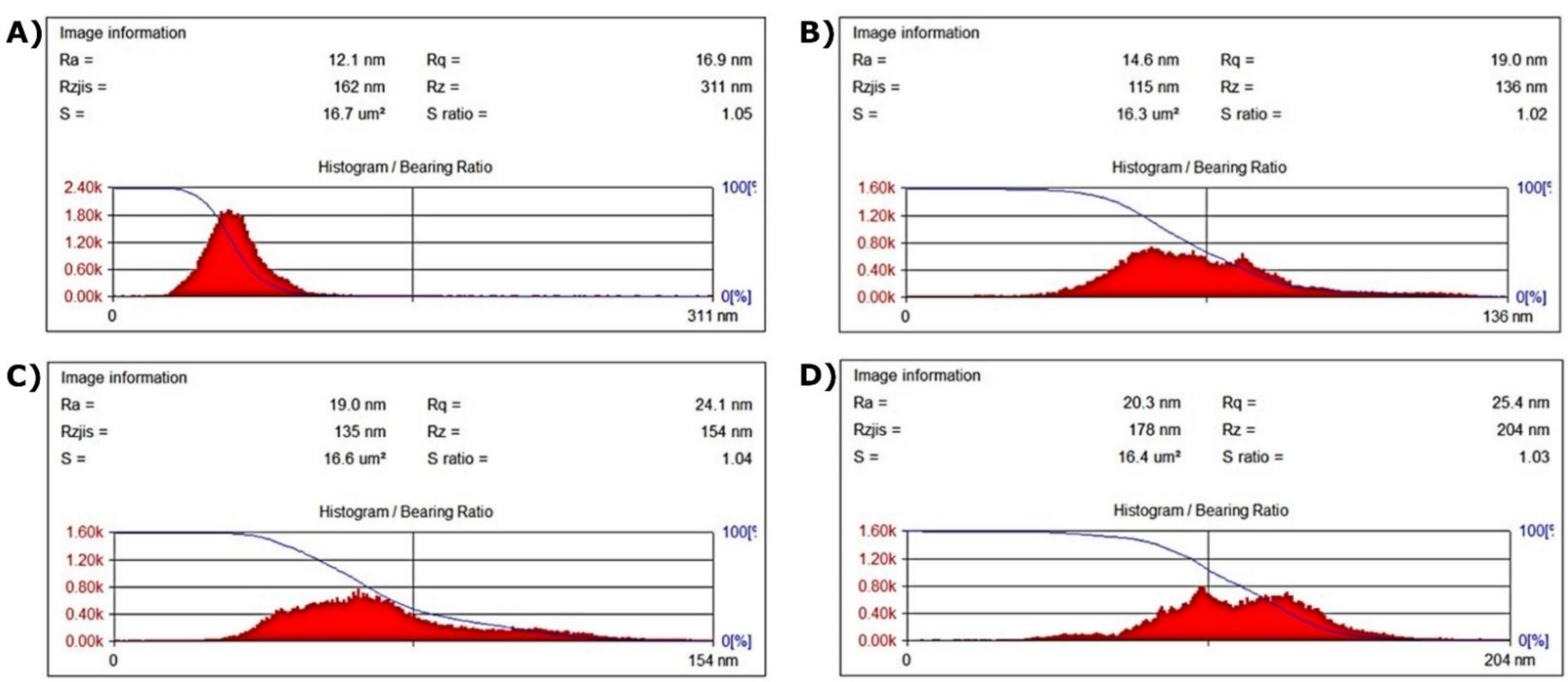


**Figure S6**. Roughness parameters and surface height distribution histograms of (**A**) **PE**, (**B**) **PE-g-MC-1-2kGy**, (**C**) **PE-g-MC-1-8kGy** and (**D**) **PE-g-MC-MeO-8kGy** films.

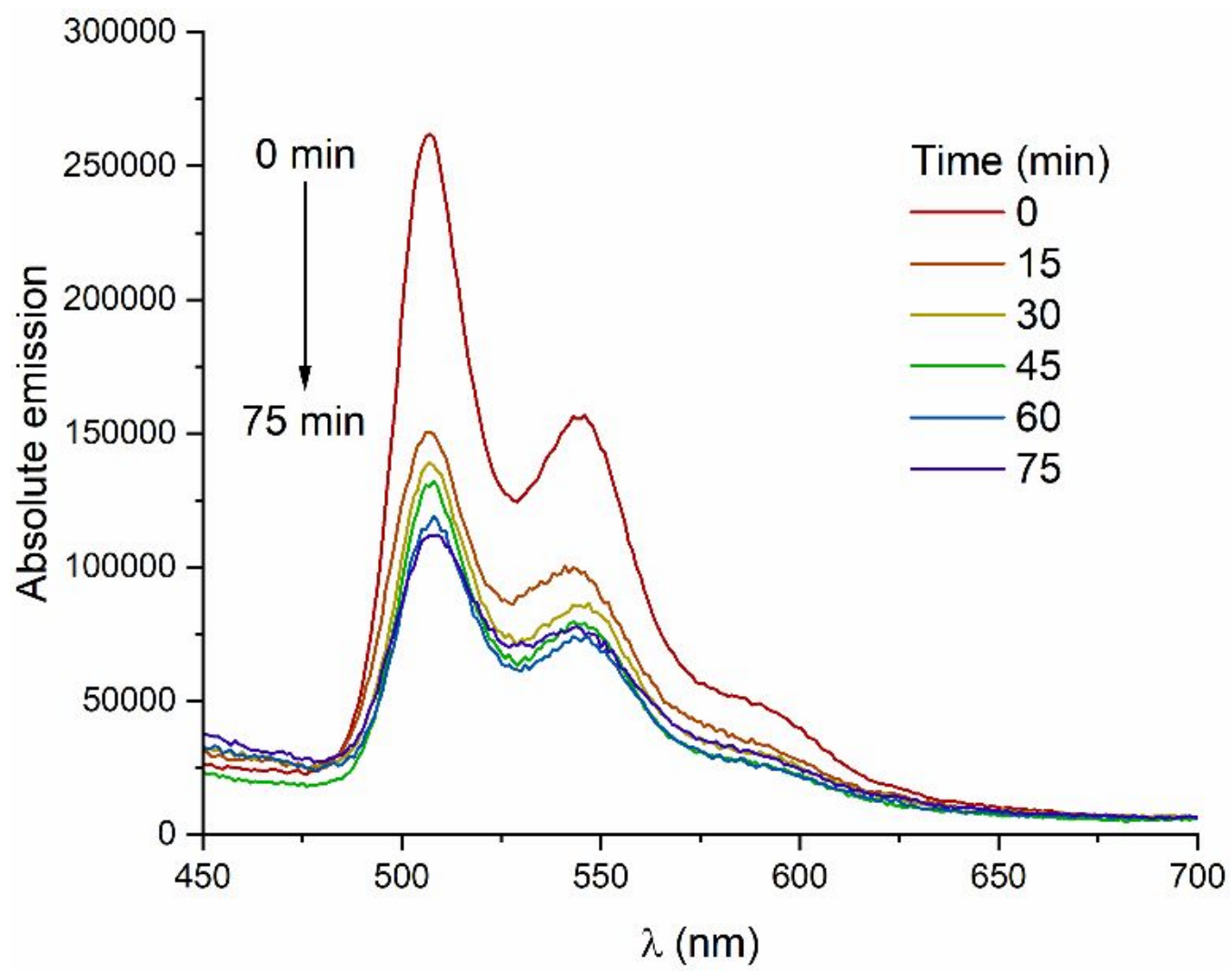


**Figure S7**. Time-dependent emission spectra ($\lambda_{ex}$ = 380 nm) of **PE-g-MC-1-8kGy** film upon treatment with NaI (50 ppm) in EtOH/$H_2O$ (1:1, v/v), recorded at 15 min intervals (0 – 75 min).

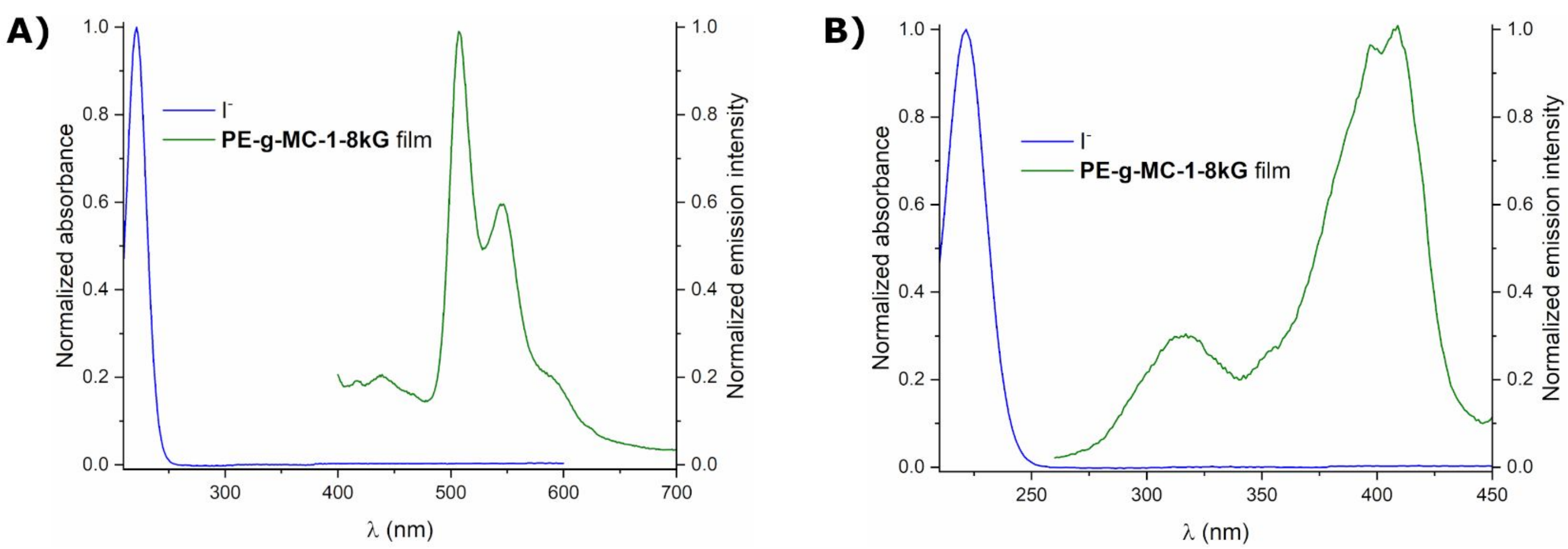


**Figure S8**. Spectral overlap analysis between the absorption of $I^-$ and (**A**) emission and (**B**) excitation spectra of **PE-g-MC-MeO-8kGy** film.

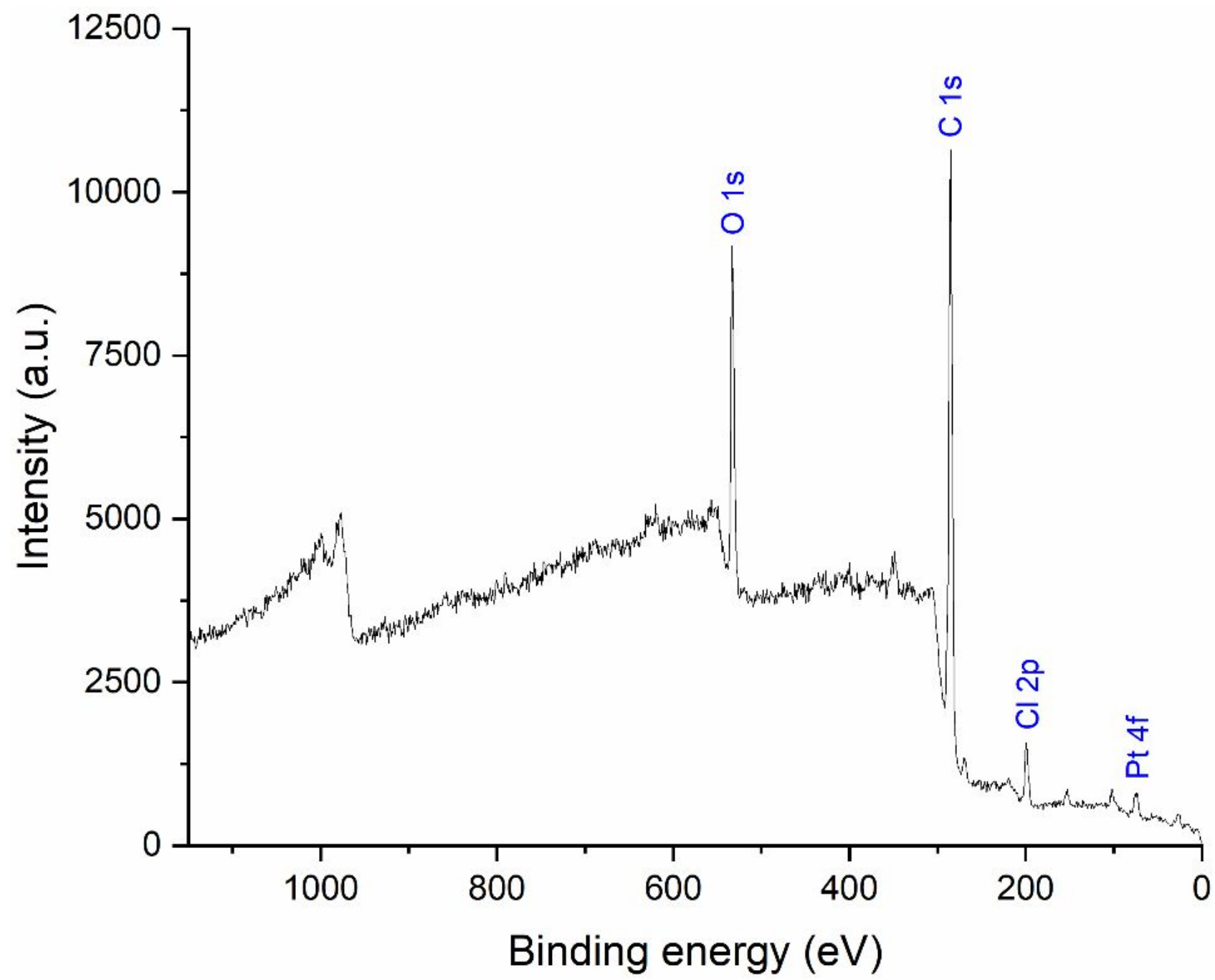


**Figure S9**. XPS survey spectrum of **PE-g-MC-1-8kGy** film upon treatment with NaI (360 ppm) in EtOH/$H_2O$ (1:1, v/v).

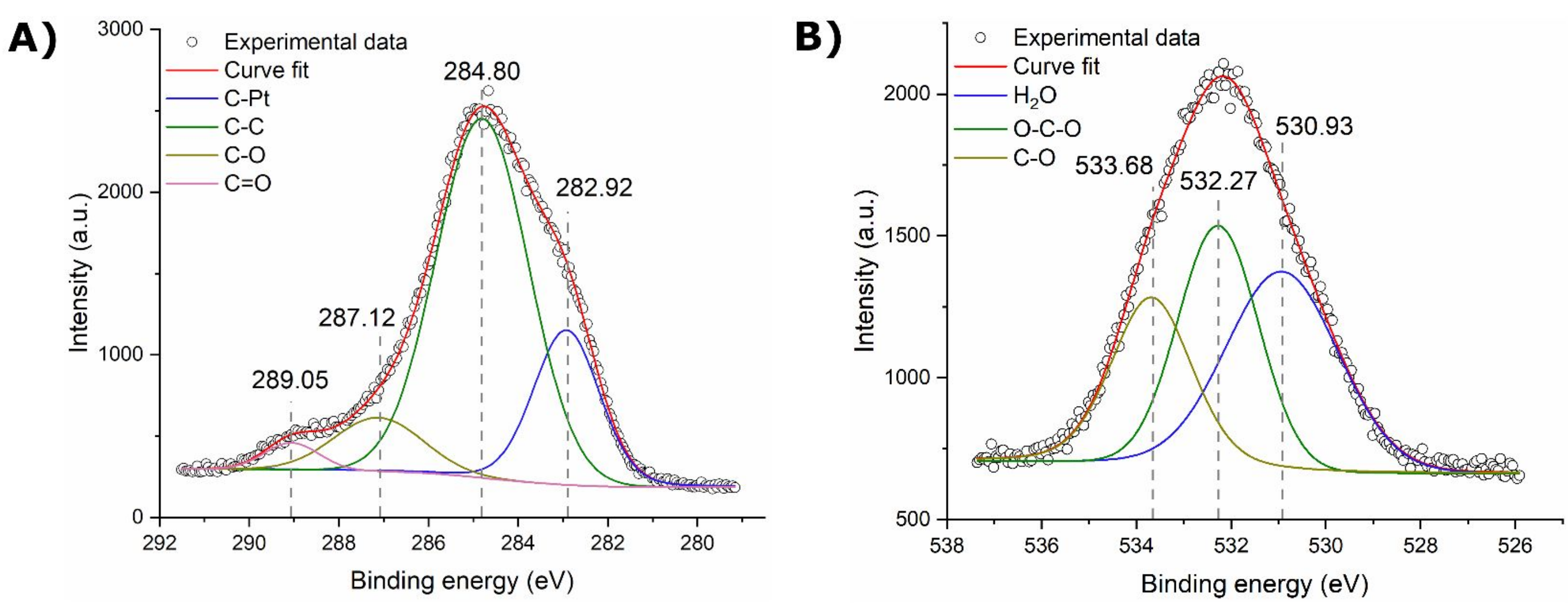


**Figure S10.** High-resolution XPS spectra of the C 1s (**A**) and O 1s (**B**) regions of **PE-g-MC-1-8kGy** after exposure to NaI (360 ppm).

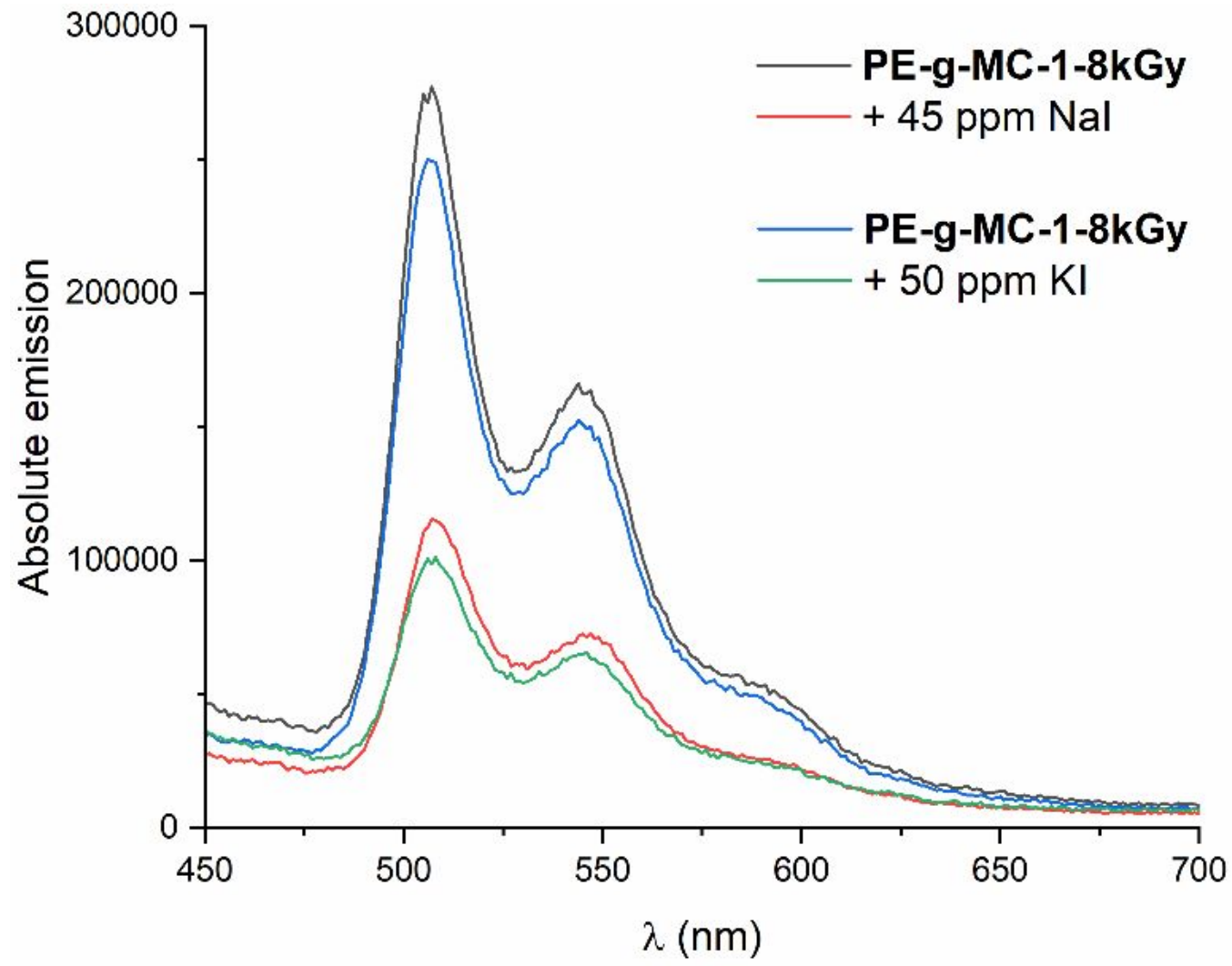


**Figure S11**. Changes in the emission spectra ($\lambda_{ex}$ = 380 nm) of **PE-g-MC-1-8kGy** film upon treatment with NaI (45 ppm) and KI (50 ppm) in EtOH/$H_2O$ (1:1, v/v).

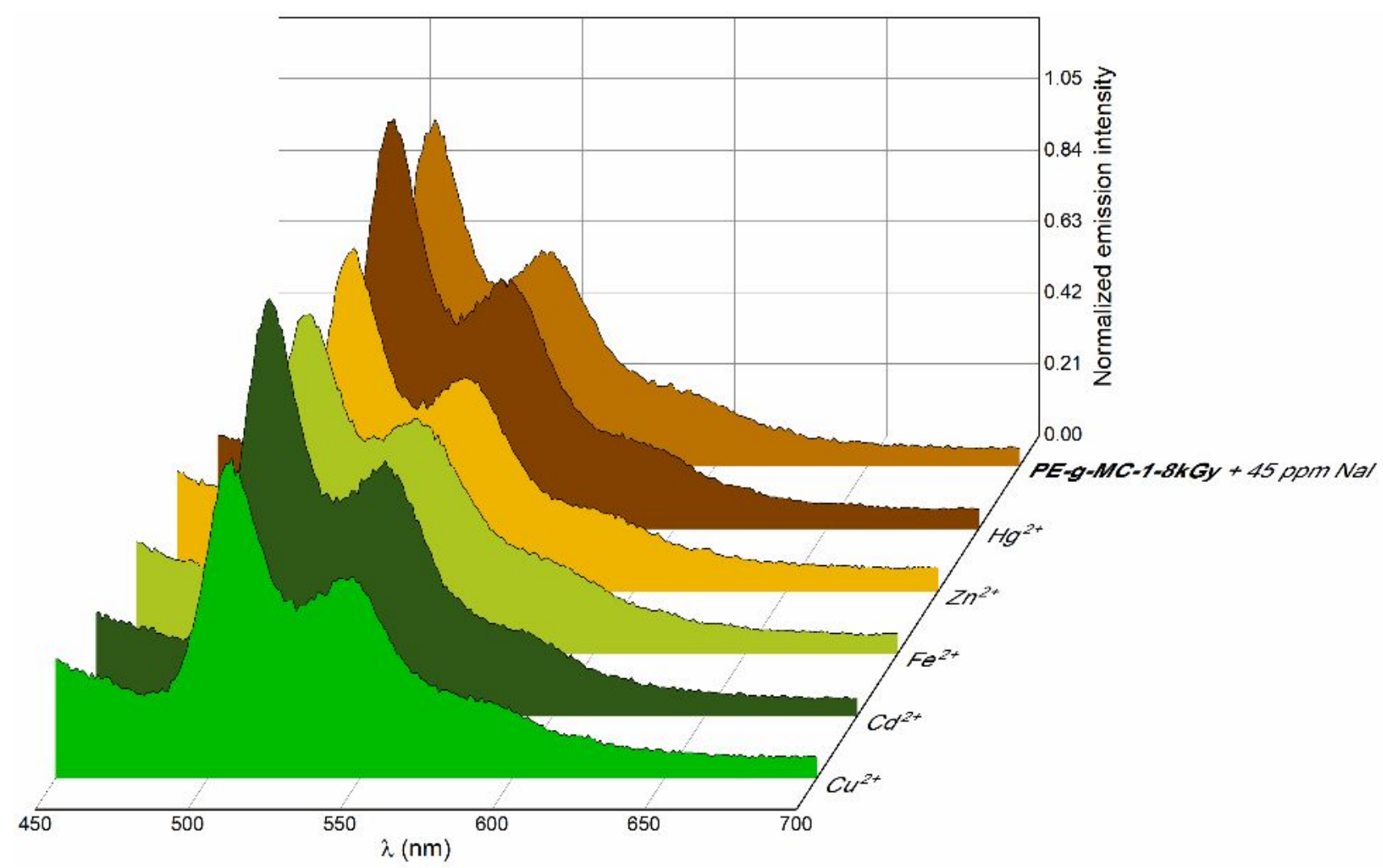


**Figure S12**. Changes in the emission spectrum ($\lambda_{ex}$ = 380 nm) of **PE-g-MC-1-8kGy** film treated with 45 ppm NaI upon addition of metal ions in EtOH/$H_2O$ (1:1, v/v) solution.

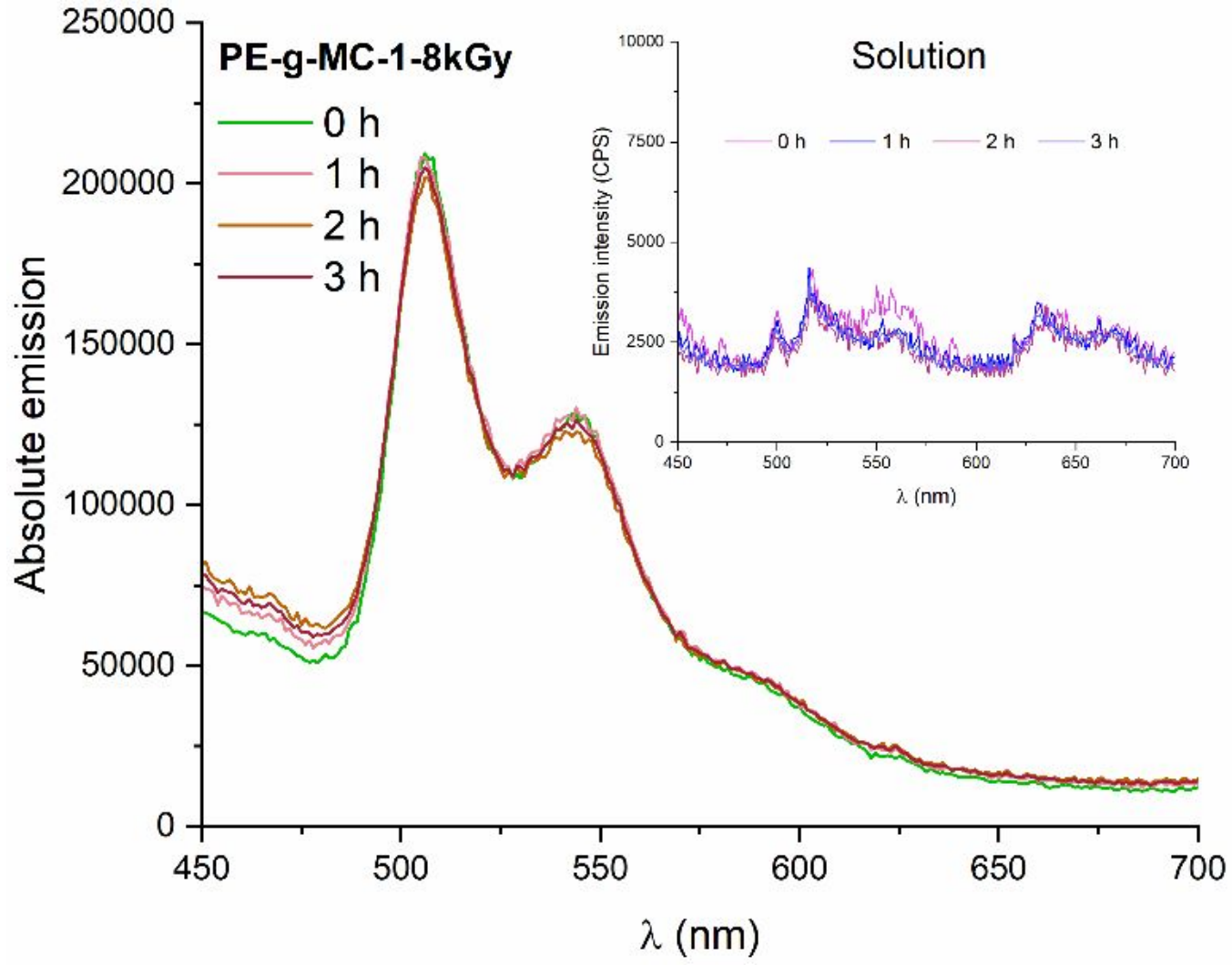


**Figure S13**. Stability of the **PE-g-MC-1-8kGy** film ($\lambda_{ex}$ = 380 nm) in EtOH/$H_2O$ (1:1, v/v) containing one drop of HCl (37 wt%) after 0, 1, 2, and 3 h. Inset: Emission spectra of the corresponding solutions collected after each exposure time.

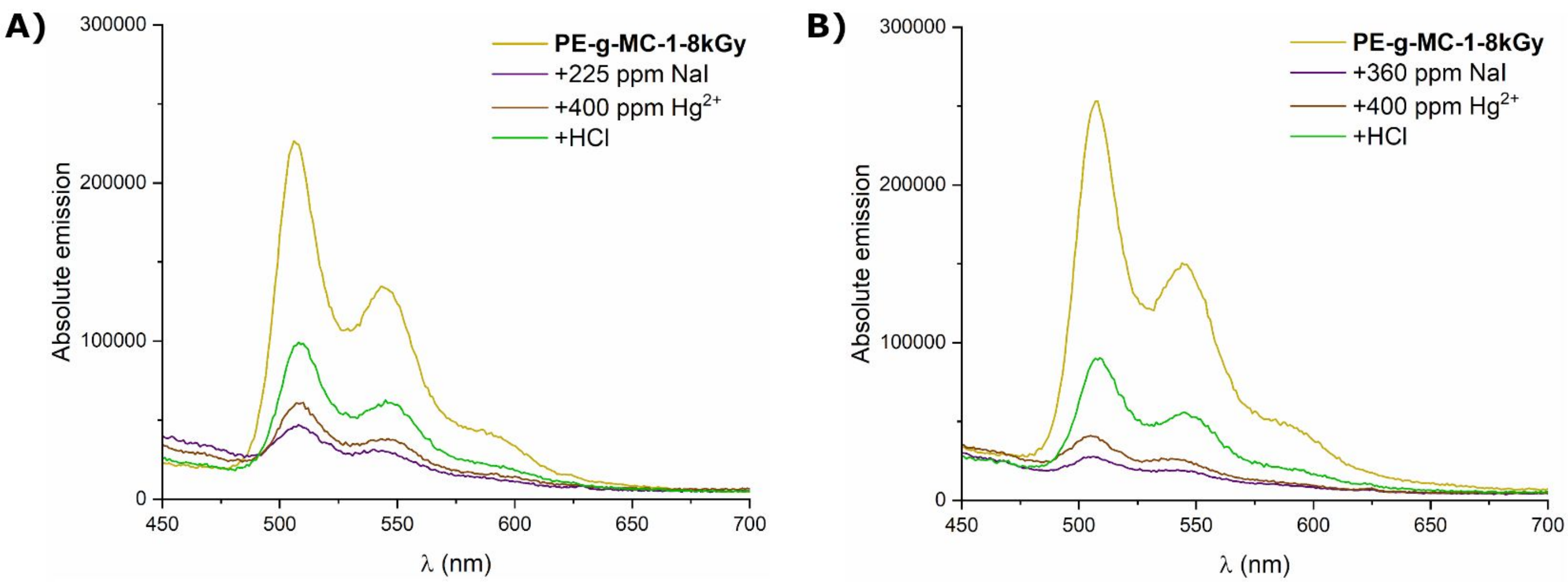


**Figure S14**. Changes in the emission spectrum ($\lambda_{ex}$ = 380 nm) of **PE-g-MC-1-8kGy** film during sequential treatments with (**A**) 225 and (**B**) 360 ppm NaI, followed by treatment with 400 ppm $Hg^{2+}$ and addition of one drop of HCl (37 wt%) in EtOH/$H_2O$ (1:1, v/v) solution.

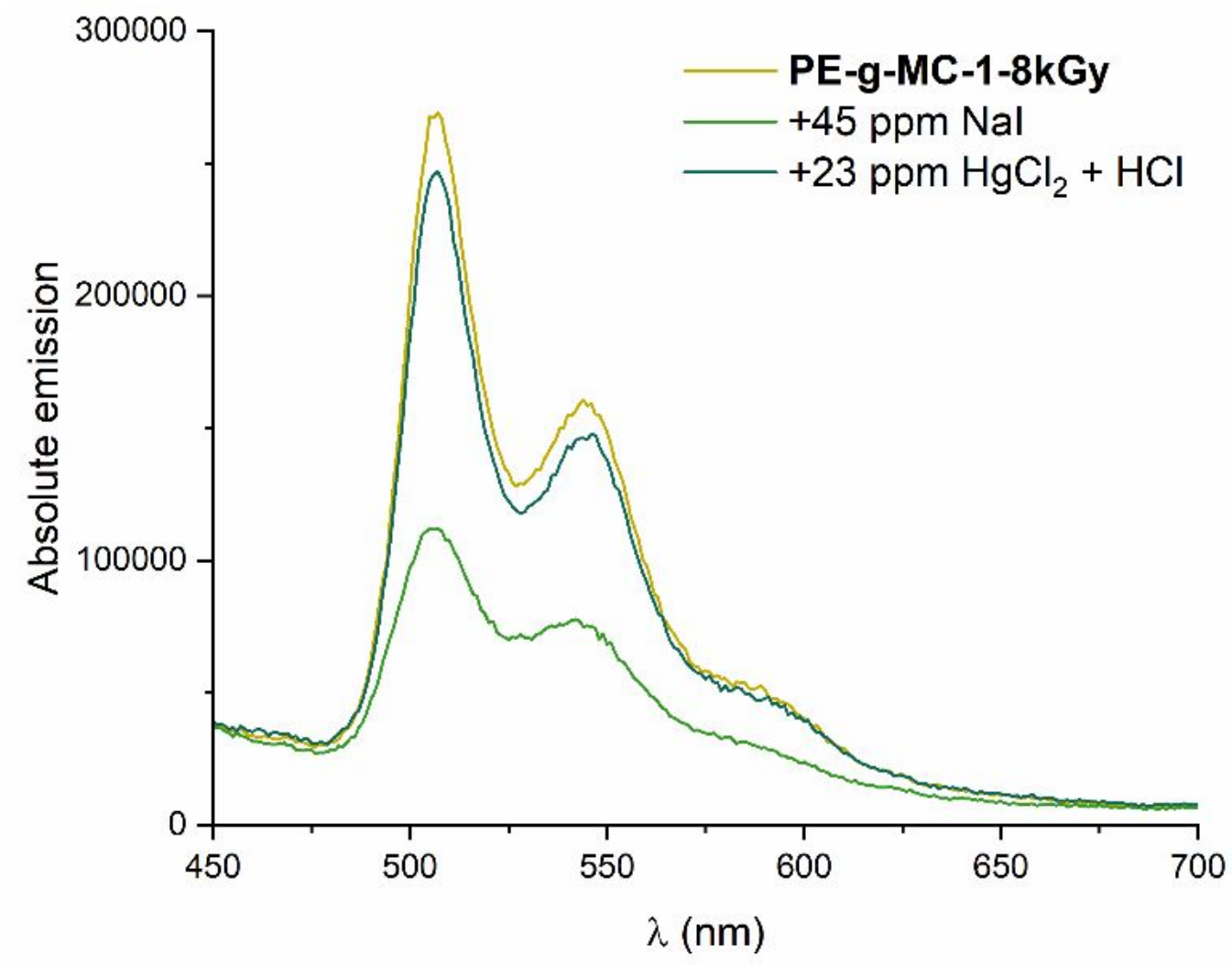


**Figure S15**. Sequential changes in the emission spectrum ($\lambda_{ex}$ = 380 nm) of **PE-g-MC-1-8kGy** film upon addition of 45 ppm NaI and 23 ppm $HgCl_2$, together with one drop of HCl (37 wt%), in EtOH/$H_2O$ (1:1, v/v) solution.

**Calculation of KI content (mg) per tablet**

290 mg Tablet —(7.4 g analyzed tablet)→ Volumetric Flask (VF) = 15 mL —(Aliquot = 5 mL)→ Vial = 10 mL

n = 3 for **A**, **B** and **C** (triplicate measurements)

Determination of $I^-$ ppm from the linear regression shown in **Figure 14**.

$$[I^-]_{ppm} = \frac{{}^{I}/_{I_o} - 0.956}{0.037\ ppm^{-1}}$$

where $I$ and $I_o$ are the emission intensities of the **PE-g-MC-1-8kGy** film after and before exposure to the dietary supplementary, respectively. Determined $[I^-]_{ppm}$ in **A**, **B** and **C** were 20.7, 22.4 and 19.6 ppm, respectively.

$$Dilution\ factor = \frac{10\ mL_{vial}}{5\ mL_{aliquot}} = 2$$

$$[I^-]_{ppm} of\ aliquot = 2\ [I^-]_{ppm}$$

Determined $[I^-]_{ppm}$ in aliquots of **A**, **B** and **C** were 41.4, 44.8 and 39.2 ppm, respectively.

$$m_{I^-} = [I^-]_{ppm} of\ aliquot\ (Volumetric\ Flask\ (0.015\ L))$$

Determined $m_{I^-}$ in **A**, **B** and **C** were 0.621, 0.672 and 0.588 mg, respectively.

$$m_{KI} = m_{I^-}\left(\frac{166\ g\ KI}{126.9\ g\ I^-}\right)$$

Determined $m_{KI}$ in **A**, **B** and **C** were 0.812, 0.879 and 0.769 mg, respectively.

$$m_{KI,\ tablet} = m_{KI}\left(\frac{290\ mg_{tablet}}{7.4\ mg_{analyzed\ tablet}}\right)$$

Determined $m_{KI,\ tablet}$ in **A**, **B** and **C** were 31.8, 34.4 and 30.1 mg, respectively.

KI mean ($m_{KI,mean}$) and Standard Deviation (SD):

A = 31.8 mg, B = 34.4 mg and C = 30.1 mg

$$m_{KI,mean} = \frac{31.8\ mg + 34.4\ mg + 30.1\ mg}{3} = 32.1\ mg$$

$$SD = \sqrt{\frac{\Sigma(m_{KI,tablet} - m_{KI,\ mean})^2}{n-1}} = 2.1\ mg$$

Relative standard deviation (%RSD):

$$\%RSD = \left(\frac{SD}{m_{KI,mean}}\right)100 = \left(\frac{2.1\ mg}{32.1\ mg}\right)100 = 6.54\%$$

**Reproducibility data and statistical analysis**

The reproducibility and statistical analysis were performed using three circular **PE-g-MC-1-8kGy** samples. The standard deviation (SD), relative standard deviation (RSD, %), and emission recovery (%) were calculated from the three measurements obtained at each point of the sensing/regeneration cycles according to the equations below:

$$Intensity_{mean} = \frac{1}{3}\sum_{i=1}^{3} I_i$$

where $I_i$ is the emission intensity measured for sample i and i is the sample number (1 – 3).

$$SD = \sqrt{\frac{\Sigma(I_i - Intensity_{mean})^2}{n-1}}$$

$$\%RSD = \left(\frac{SD}{Intensity_{mean}}\right)100$$

$$\%Emission\ recovery = \left(\frac{Intensity_{mean}}{Intensity_{cycle=0}}\right)100$$

Determined values are listed in **Table S2**.

**Table S1.** XPS fitting parameters for the Pt 4f, Cl 2p, C 1s, and O 1s regions of **PE-g-MC-1-8kGy**.

| Region | Component[a] | Binding energy (eV) | FWHM | $X^2$ |
|---|---|---|---|---|
| Pt 4f | $4f_{5/2}$ | 75.96 | 2.43 | 1.31 |
| | $4f_{7/2}$ | 72.64 | 3.62 | |
| Cl 2p | $2p_{1/2}$ | 199.82 | 2.72 | 1.27 |
| | $2p_{3/2}$ | 198.06 | 3.20 | |
| C 1s | C=O | 289.05 | 1.44 | 1.37 |
| | C–O | 287.12 | 2.33 | |
| | C–C | 284.80 | 2.42 | |
| | C–Pt | 282.92 | 1.74 | |
| O 1s | C–O | 533.68 | 1.99 | 1.16 |
| | O–C–O | 532.27 | 1.99 | |
| | $H_2O$ | 530.93 | 2.79 | |

[a]Component assignments were based on References 1 – 3.

**Table S2.** Emission recovery (%) values and relative standard deviation (RSD) during the reusability cycles of **PE-g-MC-1-8kGy.**

| Cycle | Intensity at 507 nm | | | Mean ± SD | RSD (%) | Emission Recovery (%) |
|---|---|---|---|---|---|---|
| | n = 1 | n = 2 | n = 3 | | | |
| 0 | 261547 | 272513 | 273510 | 269190 ± 7867 | 2.92 | 100 |
| 0.5 | 106324 | 112823 | 116703 | 111950 ± 5065 | 4.52 | NC[a] |
| 1 | 240827 | 243772 | 255921 | 246840 ± 7986 | 3.23 | 91.7 |
| 1.5 | 122679 | 130569 | 132762 | 128670 ± 5188 | 4.03 | NC[a] |
| 2 | 241756 | 250246 | 251098 | 247700 ± 7285 | 2.94 | 92.0 |
| 2.5 | 121434 | 125389 | 130295 | 125706 ± 5456 | 4.34 | NC[a] |
| 3 | 235459 | 236485 | 243046 | 238330 ± 6046 | 2.53 | 88.5 |

[a]NC = Not calculated.

## REFERENCES


1. Naumkin, A. V.; Kraut-Vass, A.; Gaarenstroom, S. W.; Powell, C. J. NIST X-ray Photoelectron spectroscopy database, NIST SRD 20, Version 4.1; National Institute of Standards and Technology: Gaithersburg, MD, **2012**. https://srdata.nist.gov/xps/ (accessed August 14, 2026).

2. Thermo Fisher Scientific. Table of elements: X-ray photoelectron spectroscopy of atomic elements. https://www.thermofisher.com/mx/es/home/materials-science/learning-center/periodic-table.html (accessed August 14, 2026).

3. Biesinger, M. C. X-ray photoelectron spectroscopy (XPS) reference pages: Chlorine. XPSFitting. https://www.xpsfitting.com/search/label/Chlorine (accessed August 14, 2026).
